# Supplementary material for: Triplet maintenance therapy of olaparib, pembrolizumab and bevacizumab in women with BRCA wild-type, platinum-sensitive recurrent ovarian cancer: the multicenter, single-arm phase II study OPEB-01/APGOT-OV4
Source: Nat Commun. 2023 Sep 6;14:5476. doi: 10.1038/s41467-023-40829-2 (PMC10482952; doi:10.1038/s41467-023-40829-2)
Supplement: Supplementary file 1 — Supplementary Information [file 41467_2023_40829_MOESM1_ESM.pdf]

Supplementary Information

Triplet maintenance therapy of olaparib, pembrolizumab and bevacizumab in women with BRCA wild-type, platinum-sensitive recurrent ovarian cancer: the multi-center, single-arm phase II study OPEB-01/APGOT-OV4

Supplementary Fig. 1. Progression-free survival stratified by **a** Homologous recombination deficiency (HRD) status, **b** Response to second line chemotherapy (complete or partial response), and **c** Programmed death ligand-1 combined positive score (PD-L1 CPS) (≥ 1 positive, < 1 negative).

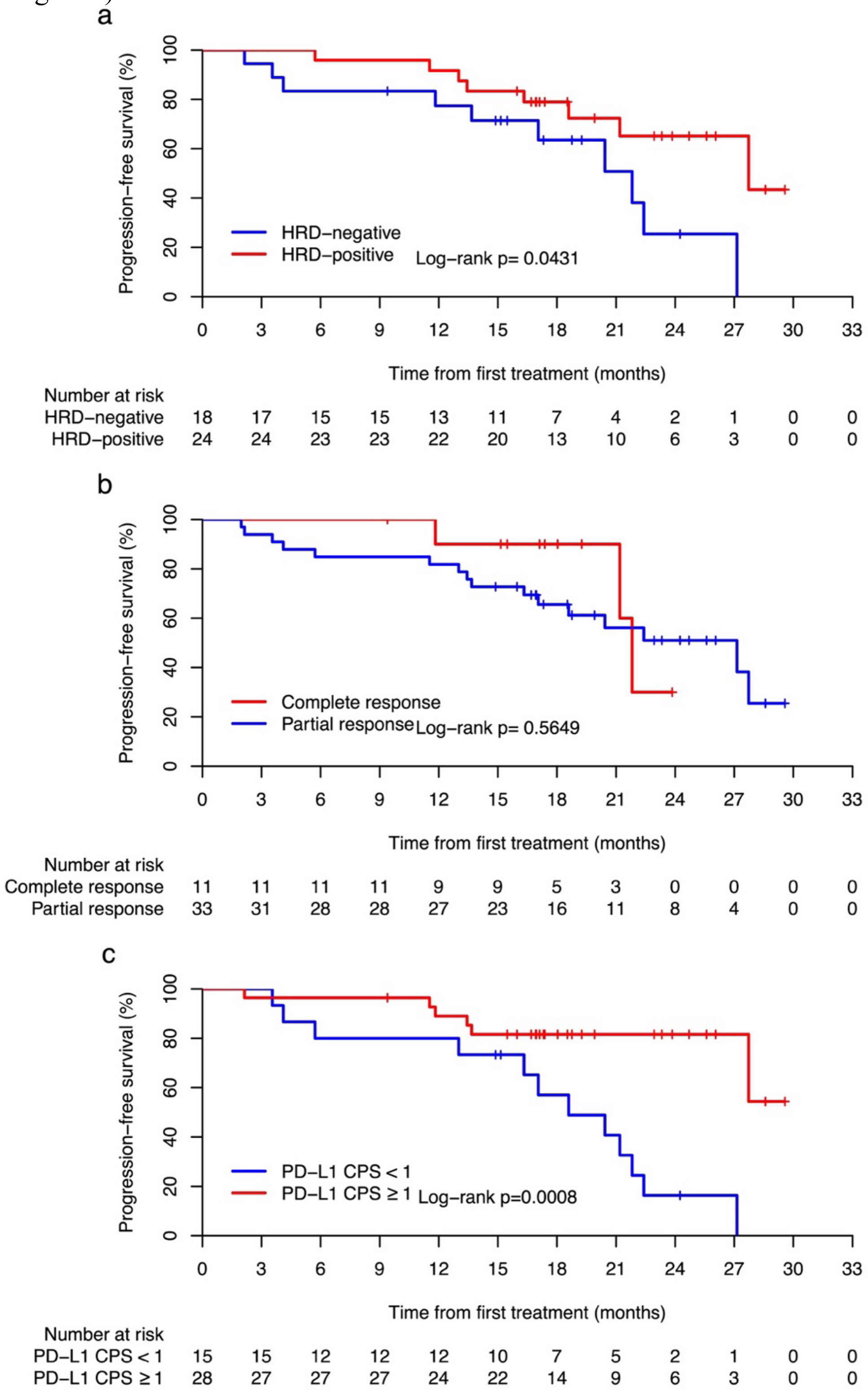

Supplementary Fig. 2. Therapy outcomes stratified by homologous recombination deficiency (HRD) and Programmed death ligand-1 combined positive score (PD-L1) status. Results are shown for those with available biomarker status.

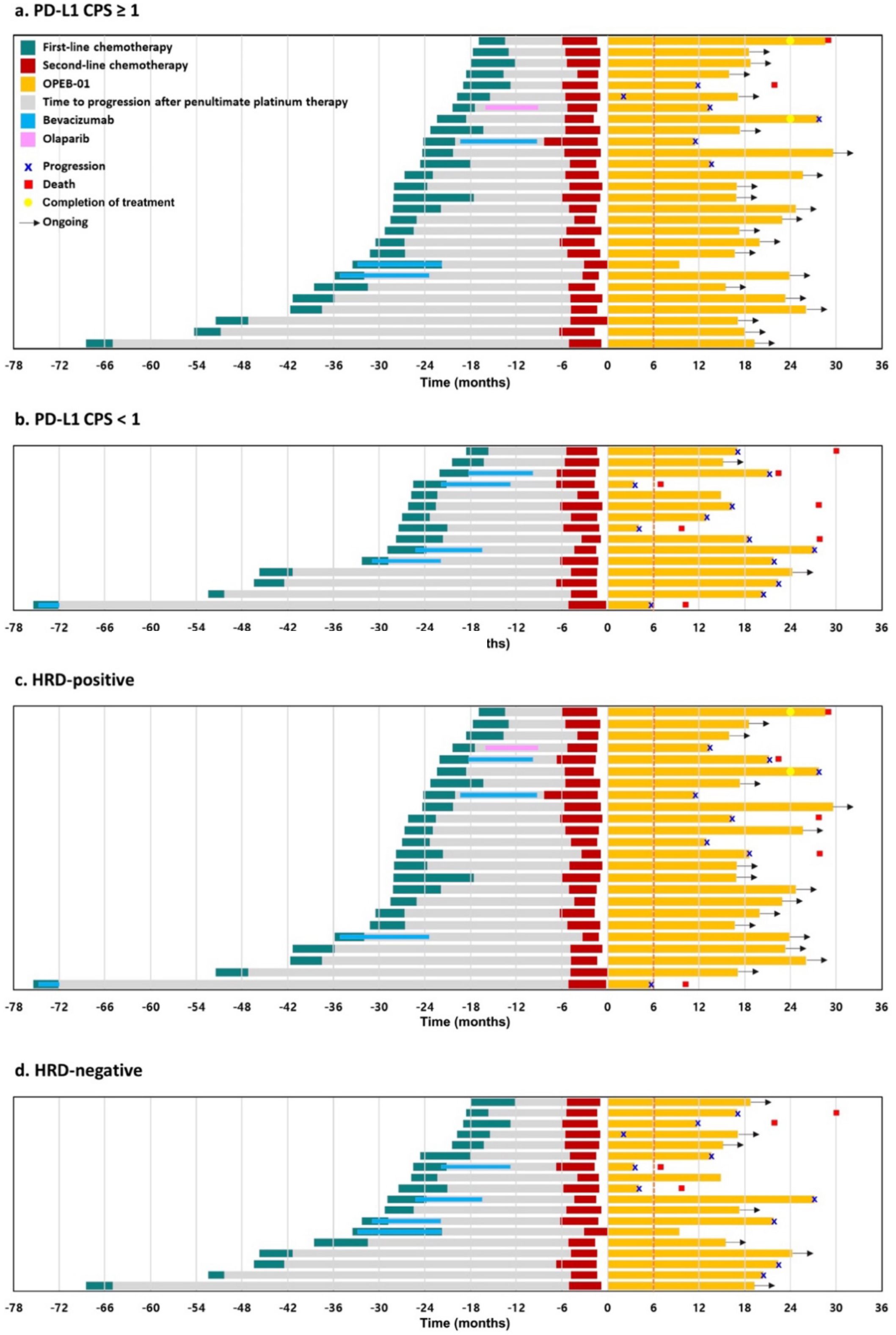

Supplementary Table 1. Summary statistics for Adverse Events (AEs).

	Patients (N, %)
All grade AEs	44 (100%)
Grade 3 or above	23 (52.3%)
Serous AEs	9 (20.5%)
AEs leading to death	0 (0%)
AEs leading to dose reduction for olaparib	27 (61.4%)
AEs leading to dose interruption	
Any of the study drugs	38 (86.4%)
Olaparib	32 (72.7%)
Pembrolizumab	34 (77.3%)
Bevacizumab	33 (75.0%)
AEs leading to dose discontinuation	
Any of the study drugs	5 (11.4%)
All three study drugs	1 (2.3%)

Supplementary Table 2. Adverse events.

	Total	Grade1	Grade2	Grade3	Grade 4
Any adverse events	44 (100%)	37 (84.1%)	43 (97.7%)	20 (45.5%)	0 (0%)
Abdominal discomfort	6 (13.6%)	2 (4.5%)	3 (6.8%)	1 (2.3%)	0 (0%)
Anemia	17 (38.6%)	0 (0%)	7 (15.9%)	10 (22.7%)	0 (0%)
Abscess	1 (2.3%)	0 (0%)	0 (0%)	1 (2.3%)	0 (0%)
Blood creatinine increase	7 (15.9%)	6 (13.6%)	1 (2.3%)	0 (0%)	0 (0%)
Chest discomfort	3 (6.8%)	2 (4.5%)	1 (2.3%)	0 (0%)	0 (0%)
Constipation	3 (6.8%)	1 (2.3%)	2 (4.5%)	0 (0%)	0 (0%)
COVID-19	9 (20.5%)	5 (11.4%)	4 (9.1%)	0 (0%)	0 (0%)
Decreased appetite	10 (22.7%)	5 (11.4%)	5 (11.4%)	0 (0%)	0 (0%)
Dyspepsia	25 (56.8%)	2 (4.5%)	22 (50.0%)	1 (2.3%)	0 (0%)
Eosinophilia	4 (9.1%)	1 (2.3%)	1 (2.3%)	2 (4.5%)	0 (0%)
Epistaxis	5 (11.4%)	4 (9.1%)	1 (2.3%)	0 (0%)	0 (0%)
Gastroesophagitis	6 (13.6%)	2 (4.5%)	4 (9.1%)	0 (0%)	0 (0%)
General weakness	18 (40.9%)	3 (6.8%)	15 (34.1%)	0 (0%)	0 (0%)
Hand-foot syndrome	3 (6.8%)	1 (2.3%)	2 (4.5%)	0 (0%)	0 (0%)
Headache	3 (6.8%)	1 (2.3%)	2 (4.5%)	0 (0%)	0 (0%)
Hypertension	6 (13.6%)	3 (6.8%)	1 (2.3%)	2 (4.5%)	0 (0%)
Insomnia	3 (6.8%)	1 (2.3%)	2 (4.5%)	0 (0%)	0 (0%)
Mucositis	10 (22.7%)	1 (2.3%)	10 (22.7%)	0 (0%)	0 (0%)
Myelodysplastic syndrome	1 (2.3%)	0 (0%)	0 (0%)	0 (0%)	1 (2.3%)
Nausea	26 (59.1%)	11 (25%)	14 (31.8%)	1 (2.3%)	0 (0%)
Neutropenia	17 (38.6%)	0 (0%)	13 (29.6%)	3 (6.8%)	0 (0%)
Pain in extremity	4 (9.1%)	1 (2.3%)	3 (6.8%)	0 (0%)	0 (0%)
Platelet count decrease	4 (9.1%)	0 (0%)	2 (4.5%)	1 (2.3%)	0 (0%)
Poor oral intake	11 (25.0%)	0 (0%)	11 (25.0%)	0 (0%)	0 (0%)
Proteinuria	19 (43.2%)	3 (6.8%)	14 (31.8%)	2 (4.5%)	0 (0%)
Pyrexia	4 (9.1%)	4 (9.1%)	0 (0%)	0 (0%)	0 (0%)
Renal abscess	1 (2.3%)	0 (0%)	0 (0%)	1 (2.3%)	0 (0%)
Rhinitis	4 (9.1%)	1 (2.3%)	3 (6.8%)	0 (0%)	0 (0%)
Small bowel perforation	1 (2.3%)	0 (0%)	0 (0%)	1 (2.3%)	0 (0%)
Vomiting	4 (9.1%)	2 (4.5%)	2 (4.5%)	0 (0%)	0 (0%)
Immune-related adverse events*					
Alanine aminotransferase increase	2 (4.5%)	2 (4.5%)	1 (2.3%)	1 (2.3%)	0 (0%)
Arthralgia	6 (13.6%)	3 (6.8%)	3 (6.8%)	0 (0%)	0 (0%)
Aspartate aminotransferase increase	6 (13.6%)	4 (9.1%)	2 (4.5%)	0 (0%)	0 (0%)
Blood thyroid stimulating hormone increase	7 (15.9%)	5 (11.4%)	1 (2.3%)	1 (2.3%)	0 (0%)
Cellulitis	1 (2.3%)	0 (0%)	0 (0%)	1 (2.3%)	0 (0%)
Diabetes mellitus	1 (2.3%)	0 (0%)	0 (0%)	1 (2.3%)	0 (0%)
Diarrhea	2 (4.5%)	0 (0%)	2 (4.5%)	0 (0%)	0 (0%)
Fatigue	6 (13.6%)	4 (9.1%)	2 (4.5%)	0 (0%)	0 (0%)

Hyperglycemia	1 (2.3%)	0 (0%)	0 (0%)	0 (0%)	0 (0%)
Hyperthyroidism	6 (13.6%)	2 (4.5%)	4 (9.1%)	0 (0%)	0 (0%)
Hypophysitis	1 (2.3%)	0 (0%)	1 (2.3%)	0 (0%)	0 (0%)
Hypothyroidism	1 (2.3%)	0 (0%)	1 (2.3%)	0 (0%)	0 (0%)
Liver function test abnormal	1 (2.3%)	0 (0%)	0 (0%)	1 (2.3%)	0 (0%)
Myalgia	4 (9.1%)	2 (4.5%)	1 (2.3%)	1 (2.3%)	0 (0%)
Rash	4 (9.1%)	1 (2.3%)	2 (4.5%)	1 (2.3%)	0 (0%)
Shingles	1 (2.3%)	0 (0%)	0 (0%)	1 (2.3%)	0 (0%)
Stomatitis	5 (11.4%)	1 (2.3%)	4 (9.1%)	0 (0%)	0 (0%)
Thyroiditis	9 (20.5%)	6 (13.6%)	3 (6.8%)	0 (0%)	0 (0%)

Data are presented as n (%). Any grade event occurring in at least three patients (7.7%) and all grade 3 and 4 adverse events are shown. No death due to adverse event was reported. *Immune-mediated adverse events of at least one event are shown; the designation of these events as immune-mediated was at the discretion of the investigator.

Supplementary Table 3. Study scheme.

Drug	Treatment protocol
Olaparib	300mg BID (oral); during each treatment cycle for up to 2 years*
Pembrolizumab	200mg Q3W (intravenous); day 1 of each 3-week cycle from cycle 2 through cycle 35
Bevacizumab	15mg/kg Q3W (intravenous); day 1 of each 3-week cycle until PD

Abbreviations: OC, ovarian cancer; ECOG, Eastern Cooperative Oncology Group; CR, complete response; PR, partial response; PD, progressive disease; Q3W, every 3 weeks; BID, twice daily

* Patients who had a partial response at 2 years were permitted to continue receiving treatment.

Supplementary Note

SPONSOR: Jung-Yun Lee

TITLE: A single-arm phase II study of Olaparib maintenance with Pembrolizumab & Bevacizumab in BRCA non-mutated patients with platinum-sensitive recurrent ovarian cancer (OPEB-01)

1.0 TRIAL SUMMARY

Abbreviated Title	OPEB-01
Trial Phase	*Phase II*
Clinical Indication	Platinum-sensitive recurrent BRCA wild type ovarian cancer
Trial Type	Single arm
Type of control	n/a
Route of administration	Olaparib for po / pembrolizumab for iv / bevacizumab for iv
Trial Blinding	n/a
Treatment Groups	Maintenance : Olaparib 300mg (twice daily [BID])<br>Pembrolizumab 200mg every 3 weeks (Q3W) from 2nd infusion for up to 35 infusions<br>Bevacizumab 15mg/kg or 7.5mg/kg every 3 weeks (Q3W)
Number of trial participants	44
Estimated enrollment period	*12 months*
Estimated duration of trial	*72 months*
Duration of Participation	60 months
Estimated average length of treatment per patient	12 months

2.0 TRIAL DESIGN

2.1 Trial Design

The study design is depicted in Figure 1.

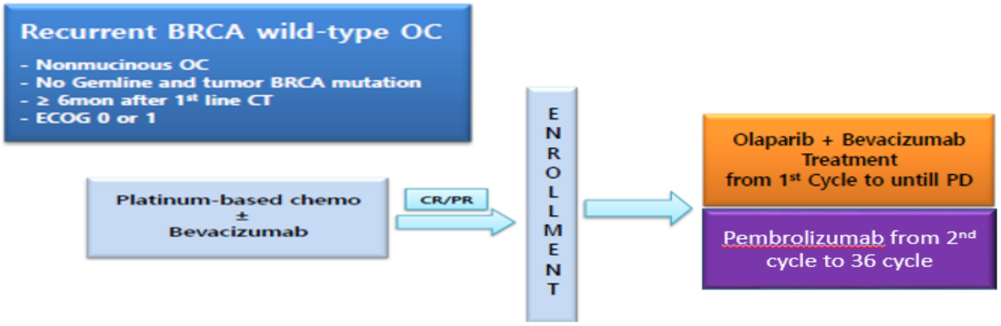

Figure 1. Study diagram

Abbreviations: OC, ovarian cancer; CT, chemotherapy; CR, complete remission; PR, partial remission

2.2 Trial Diagram

The study diagram is depicted in Figure 2.

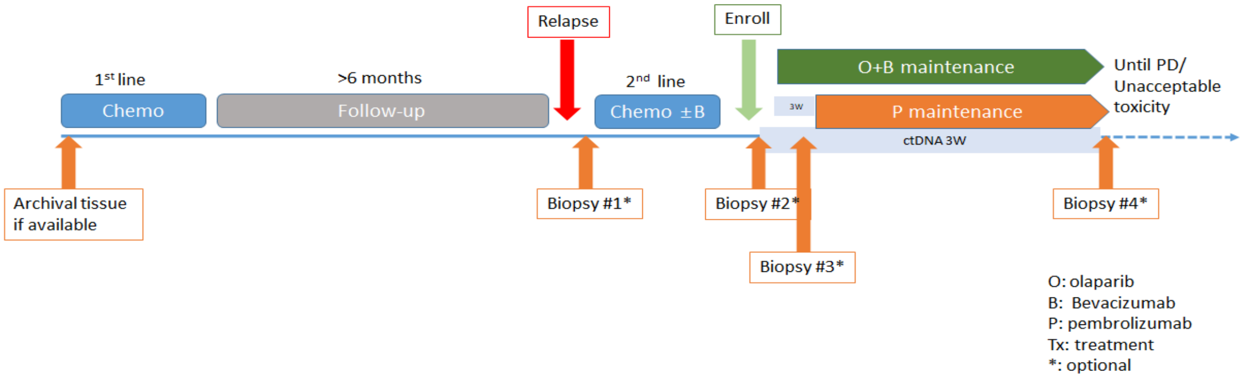

Figure 2. Study diagram

Abbreviations: Chemo, Chemotherapy; CR, complete remission; PR, partial remission; O, Olaparib; B, bevacizumab; P, pembrolizumab; PD, progressive disease

3.0 OBJECTIVE(S) & HYPOTHESIS(ES)

3.1 Primary Objective(s) & Hypothesis(es)

(1) **Objective:** To determine the efficacy of Olaparib maintenance with Bevacizumab & Pembrolizumab by assessment of progression-free survival (6 months PFS rate)

(2) **Hypothesis:** Olaparib maintenance with Bevacizumab & Pembrolizumab is synergistic in BRCA wild-type patients with platinum-sensitive recurrent ovarian cance

3.2 Secondary Objective(s) & Hypothesis(es)

(1) Objective: To determine the efficacy of Olaparib maintenance with Bevacizumab & Pembrolizumab by assessment of Overall survival, progression-free survival rate, Time to progression, Time to first subsequent treatment (or death), Time to second subsequent treatment, PFS2

(2) Safety objective: To evaluate the safety and tolerability of Olaparib maintenance with Bevacizumab & Pembrolizumab

3.3 Exploratory Objective

(1) **Objective:** To identify molecular biomarkers that may be indicative of clinical response/resistance, safety, and/or the mechanism of action of pembrolizumab, olaparib, and bevacizumab

4.0 BACKGROUND & RATIONALE

4.1 Background

4.1.1 Pembrolizumab

Pembrolizumab is a potent humanized immunoglobulin G4 (IgG4) monoclonal antibody (mAb) with high specificity of binding to the programmed cell death 1 (PD-1) receptor, thus inhibiting its interaction with programmed cell death ligand 1 (PD-L1) and programmed cell death ligand 2 (PD-L2). Based on preclinical in vitro data, pembrolizumab has high affinity and potent receptor blocking activity for PD-1. Pembrolizumab has an acceptable preclinical safety profile and is in clinical development as an intravenous (IV) immunotherapy for advanced malignancies. Keytruda® (pembrolizumab) is indicated for the treatment of patients across a number of indications because of its mechanism of action to bind the PD-1 receptor on the T cell. For more details on specific indications refer to the Investigator brochure (IB).

4.1.2 Olaparib

Olaparib is a potent PARP inhibitor (PARP-1, -2 and -3) that is being developed as an oral therapy, both as a monotherapy (including maintenance) and for combination with chemotherapy and other anti-cancer agents. PARP inhibition is a novel approach to targeting tumors with deficiencies in DNA repair mechanisms. PARP enzymes are essential for repairing DNA single-strand breaks (SSBs). Inhibiting PARPs leads to the persistence of SSBs, which are then converted to the more serious DNA double-strand breaks (DSBs) during the process of DNA replication. During the process of cell division, DSBs can be efficiently repaired in normal cells by homologous recombination repair (HRR). Tumors with HRR deficiencies (HRD), such as OCs in patients with BRCA1/2 mutations, cannot accurately repair the DNA damage, which may become lethal to cells as it accumulates. In such tumor types, olaparib may offer a potentially efficacious and less toxic cancer treatment compared with currently available chemotherapy regimens.
Refer to the IB/approved labeling for detailed background information on olaparib.

4.1.3 Pharmaceutical and Therapeutic Background

4.1.3.1 Inhibitor of PD-1 as a Target for Cancer

The importance of intact immune surveillance function in controlling outgrowth of neoplastic transformations has been known for decades [Disis, 2010]. Accumulating evidence shows a correlation between tumor-infiltrating lymphocytes in cancer tissue and favorable prognosis in various malignancies. In particular, the presence of CD8+ T-cells and the ratio of CD8+ effector T-cells/FoxP3+ regulatory T-cells (T-regs) correlates with improved prognosis and long-term survival in solid malignancies, such as ovarian, colorectal, and pancreatic cancer; hepatocellular carcinoma; malignant melanoma; and renal cell carcinoma. Tumor-infiltrating lymphocytes can be expanded ex vivo and reinfused, inducing durable objective tumor responses in cancers such as melanoma [Dudley et al., 2005; Hunder et al., 2008].

The PD-1 receptor-ligand interaction is a major pathway hijacked by tumors to suppress immune control. The normal function of PD-1, expressed on the cell surface of activated T-cells under healthy conditions, is to down-modulate unwanted or excessive immune responses, including autoimmune reactions. PD-1 (encoded by the gene *Pdcd1*) is an immunoglobulin (Ig) superfamily member related to cluster of differentiation 28 (CD28) and cytotoxic T-lymphocyte-associated protein 4 (CTLA-4) that has been shown to negatively regulate antigen receptor signaling upon engagement of its ligands (PD-L1 and/or PD-L2) [Greenwald et al., 2005; Okazaki et al., 2001].

The structure of murine PD-1 has been resolved [Zhang et al., 2004]. PD-1 and its family members are type I transmembrane glycoproteins containing an Ig-variable–type (IgV-type) domain responsible for ligand binding and a cytoplasmic tail responsible for the binding of signaling molecules. The cytoplasmic tail of PD-1 contains 2 tyrosine-based signaling motifs, an immunoreceptor tyrosine-based inhibition motif, and an immunoreceptor tyrosine-based switch motif. Following T-cell stimulation, PD-1 recruits the tyrosine phosphatases, SHP-1 and SHP-2, to the immunoreceptor tyrosine-based switch motif within its cytoplasmic tail, leading to the dephosphorylation of effector molecules such as CD3 zeta (CD3ζ), protein kinase C-theta (PKCθ), and zeta-chain-associated protein kinase (ZAP70), which are involved in the CD3 T-cell signaling cascade [Okazaki et al., 2001; Chemnitz et al., 2004; Sheppard et al., 2004; and Riley, 2009]. The mechanism by which PD-1 down-modulates T-cell responses is similar to, but distinct from, that of CTLA-4, because both molecules regulate an overlapping set of signaling proteins [Parry et al., 2005; Francisco, 2010]. As a consequence, the PD-1/PD-L1 pathway is an attractive target for therapeutic intervention in ovarian cancer.

4.1.3.2 Inhibitor of PARP as a Target for Cancer

PARP1 and PARP2 are zinc-finger DNA-binding enzymes that play a critical role in DNA repair [Ame, J. C., et al 2004] by sensing DNA damage and converting it into intracellular signals that activate the base excision repair and SSB repair pathways. When a break in DNA occurs, PARP enzymes are recruited to and bind at the end of the broken DNA strands, activating their enzymatic activity. PARP subsequently catalyzes the addition of long polymers of ADP-ribose onto several other proteins associated with chromatin (eg, PARP, histones, DNA repair proteins), resulting in chromatin relaxation, rapid recruitment of DNA repair proteins, and efficient repair of the break. Under normal conditions, HRR is the preferred pathway for repairing DNA damage as it is associated with a lower rate of errors compared with other forms of DNA repair [Prakash, R., et al 2015]. During DNA replication (S phase), pre-existing SSBs are converted to DSBs as the replication machinery passes [Fong, Peter C., et al 2009], which are ultimately repaired by HRR. Cells unable to perform HRR (eg, due to inactivation of genes required for the process, such as BRCA1 or BRCA2) are more likely to use the error-prone nonhomologous end-joining (NHEJ) or alternative NHEJ pathways to repair these DSBs and are at risk for accumulating multiple lesions or loss of heterozygosity. Over time, the buildup of excessive DNA errors in combination with the inability to complete S phase (because of stalled replication forks) contributes to cell death. Clinical studies have shown that PARP inhibitors are most effective in patients with recurrent OC who are either platinum-sensitive or platinum-resistant and harbor mutations of BRCA1/2 [George, A., et al 2017]. PARP inhibitors have demonstrated effectiveness in patients without germline BRCAmut (gBRCAmut), although the magnitude has been smaller [Fong, Peter C., et al 2009]

[Gelmon, K. A., et al 2011] [Ledermann, J., et al 2012]. For example, median progression-free survival (PFS) was significantly longer for patients with platinum-sensitive, relapsed OC and gBRCAmut who were treated with olaparib as maintenance therapy compared with placebo (19.1 months versus 5.5 months; hazard ratio [HR]: 0.30, p<0.001) [Pujade-Lauraine, E., et al 2017]. Similarly, median PFS was significantly longer in patients with platinum-sensitive, relapsed OC and any BRCA1/2 status compared with placebo (8.4 months versus 4.8 months; HR: 0.35, p<0.001). Additionally, these patients had a median overall survival (OS) of 29.8 months compared with 27.8 months for placebo (HR: 0.73) [Ledermann, J., et al 2012]. In summary, treatment with PARP1/2 inhibitors represents a novel opportunity to selectively kill a subset of cancer cell types by exploiting their deficiencies in DNA repair. Human cancers exhibit genomic instability and an increased mutation rate due to underlying defects in DNA repair. These deficiencies render cancer cells more dependent on the remaining DNA repair pathways, and targeting these pathways is expected to a much greater impact on the survival of the tumor cells than on normal cells.

4.1.4 Preclinical and Clinical Trial Data

Refer to the Investigator's Brochure for Preclinical and Clinical data.

4.2 Rationale

4.2.1 Rationale for the Trial and Selected Population

4.2.1.1 Unmet needs in Platinum-sensitive recurrent ovarian cancer

Platinum-based chemotherapy is standard of care (SOC) for platinum-sensitive recurrent ovarian cancer. Olaparib maintenance therapy is very effective option only for BRCA mutated patients with platinum-sensitive recurrence. Based on study 19, Korean FDA has permitted the use of olaparib for maintenance therapy in BRCA mutated patients with platinum-sensitive recurrent ovarian cancer since 2017. For BRCA wild-type patients, few options for maintenance therapy are suggested in Korea. Bevacizumab is the one of maintenance therapy option for BRCA wild-type ovarian cancer. Korean FDA has permitted the use of bevacizumab for concurrent and maintenance use for all comers in platinum-sensitive recurrence. Based on GOG 213, median PFS after second-line chemotherapy is 6 months even with bevacizumab maintenance. Thus, there is an unmet medical need for therapies that, when used in combination with the current SOC, significantly increases the proportions of patients with complete remission and prevents disease recurrences in patients with advanced EOC.

4.2.1.2 Rationale for combination

Single agent PARP inhibitor has less activity in BRCA wild type and HR proficient tumors. Single agent Immune checkpoint inhibitor has strong activity only in small population such as PD-L1 positive and/or dMMR patients. In order to expand current indication and apply to biomarker negative patients, combination strategies are suggested.
PARP inhibitor+anti-angiogenic agents: Joyce Liu et al. shows synergistic effects of olaparib and cediranib in BRCA wild type patients [Liu et al., 2014]. AVANOVA randomized phase II study

shows the combination of niraparib and bevacizumab significantly improve PFS than niraparib alone [Mirza et al., 2019]. This survival benefit was found in non-BRCA mut and/or HRD negative patients as well as BRCA and/or HRD positive group. PAOLA randomized phase III study is evaluating synergistic effects of olaparib and bevacizumab [Ray-Coquard et al., 2019].

PARP inhibitor + Immune checkpoint inhibitor: PARP inhibitor treatment lead to an accumulation of cytosolic dsDNA which activates the cGAS-STING-TBK1-IRF3 innate immune axis; thus promoting antitumor immunity. This can be further enhanced by the introduction of immune checkpoint blockade. MEDIOLA study shows synergistic effects of olaparib and durvalumab. TOPACIO study shows promising effects of combination between niraparib and pembrolizumab regardless of BRCA/ HRD status in platinum-resistant setting [Konstantinopoulos et al., 2019].

Anti-angiogenic agents + IO agents: combination between lenvatinib+pembrolizumab shows activity in advanced endometrial cancer regardless of MSI status (ORR 24wks = 50%) [Makker et al., 2019].

In addition, safety was established for following combinations: olaparib+pembrolizumab, bevacizumab+pembrolizumab, olaparib+bevacizumab

Combination of three agents: The efficacy of combination of two agents has been evaluated through many clinical trials in ovarian cancer. We have experienced that combination therapy has synergistic in BRCA wild-type patients. At present, several randomized trials are ongoing, including NCT02484404 (olaparib, cediranib, durvalumab), DUO-O (olaparib, durvalumab, bevacizumab), ENGOT-ov43 (olaparib, pembrolizumab, bevacizumab). While clinical trials with three agents are already ongoing, it is unknown yet whether combination of three agents (PARP inhibitor, anti-angiogenic agents, and immunotherapy) has synergistic and safe for biomarker negative patients such as BRCA wild-type.

It is unknown whether BRCA wild-type patients could derive clinical benefit from olaparib maintenance with other novel agents. We suggest olaparib maintenance with bevacizumab & pembrolizumab to show synergistic effects in BRCA wild-type patients with platinum sensitive recurrence.

4.2.2 Justification for Dose

4.2.2.1 Rationale for Pembrolizumab Dosing regimen

The planned dose of pembrolizumab for this study is 200 mg every 3 weeks (Q3W). Based on the totality of data generated in the Keytruda development program, 200 mg Q3W is the appropriate dose of pembrolizumab for adults across all indications and regardless of tumor type. As outlined below, this dose is justified by:

- Clinical data from 8 randomized studies demonstrating flat dose- and exposure-efficacy relationships from 2 mg/kg Q3W to 10 mg/kg every 2 weeks (Q2W),

- Clinical data showing meaningful improvement in benefit-risk including overall survival at 200 mg Q3W across multiple indications, and
- Pharmacology data showing full target saturation in both systemic circulation (inferred from pharmacokinetic [PK] data) and tumor (inferred from physiologically-based PK [PBPK] analysis) at 200 mg Q3W

Among the 8 randomized dose-comparison studies, a total of 2262 participants were enrolled with melanoma and non-small cell lung cancer (NSCLC), covering different disease settings (treatment naïve, previously treated, PD-L1 enriched, and all-comers) and different treatment settings (monotherapy and in combination with chemotherapy). Five studies compared 2 mg/kg Q3W versus 10 mg/kg Q2W (KN001 Cohort B2, KN001 Cohort D, KN002, KN010, and KN021), and 3 studies compared 10 mg/kg Q3W versus 10 mg/kg Q2W (KN001 Cohort B3, KN001 Cohort F2 and KN006). All of these studies demonstrated flat dose- and exposure-response relationships across the doses studied representing an approximate 5- to 7.5-fold difference in exposure. The 2 mg/kg (or 200 mg fixed-dose) Q3W provided similar responses to the highest doses studied. Subsequently, flat dose-exposure-response relationships were also observed in other tumor types including head and neck cancer, bladder cancer, gastric cancer and classical Hodgkin Lymphoma, confirming 200 mg Q3W as the appropriate dose independent of the tumor type. These findings are consistent with the mechanism of action of pembrolizumab, which acts by interaction with immune cells, and not via direct binding to cancer cells.

Additionally, pharmacology data clearly show target saturation at 200 mg Q3W. First, PK data in KN001 evaluating target-mediated drug disposition (TMDD) conclusively demonstrated saturation of PD-1 in systemic circulation at doses much lower than 200 mg Q3W. Second, a PBPK analysis was conducted to predict tumor PD-1 saturation over a wide range of tumor penetration and PD-1 expression. This evaluation concluded that pembrolizumab at 200 mg Q3W achieves full PD-1 saturation in both blood and tumor.

Finally, population PK analysis of pembrolizumab, which characterized the influence of body weight and other participant covariates on exposure, has shown that the fixed-dosing provides similar control of PK variability as weight based dosing, with considerable overlap in the distribution of exposures from the 200 mg Q3W fixed dose and 2 mg/kg Q3W dose. Supported by these PK characteristics, and given that fixed-dose has advantages of reduced dosing complexity and reduced potential of dosing errors, the 200 mg Q3W fixed-dose was selected for evaluation across all pembrolizumab protocols.

4.2.2.1 RATIONALE FOR OLAPARIB DOSING REGIMEN

The dose of olaparib used in this study is 300mg BID (tablet formation) and is the current approved dose

4.2.2.2 Rationale for Bevacizumab Dosing Regimen

The dose of bevacizumab is allowed at the investigator’s discretion, where approved for the second-line treatment of EOC, as per the local SOC and approved product label.

4.2.3 Rationale for Endpoints

4.2.3.1 Efficacy Endpoints

This study will use primary endpoint of 6 months PFS rate as in figure 1.

This study will use PFS based on RECIST 1.1 criteria as assessed by the investigator as the primary endpoint. PFS is an acceptable measure of clinical benefit for a late stage study that demonstrates superiority of a new antineoplastic therapy, especially if the magnitude of the effect is large and the therapy has an acceptable risk/benefit profile. OS has been recognized as the gold standard for the demonstration of superiority of a new antineoplastic therapy.

4.2.3.2 Biomarker Research

Cancer immunotherapies represent an important and novel class of antitumor agents. However, the mechanism of action of these exciting new therapies is not completely understood and much remains to be learned regarding how best to leverage these new drugs in treating patients. Thus, to aid future patients, it is important to investigate the determinants of response or resistance to cancer immunotherapy and other treatments administered, as well as determinants of AEs in the course of our clinical studies. These efforts may identify novel predictive/PD biomarkers and generate information that may better guide single-agent and combination therapy with immuno-oncology drugs. To identify novel biomarkers, biospecimens (ie, blood components, tumor material) will be collected to support analyses of cellular components (eg, protein, DNA, RNA, metabolites) and other circulating molecules. Investigations may include but are not limited to:Germline (blood) genetic analyses (eg, SNP analyses, whole exome sequencing, whole genome sequencing)
This research may evaluate whether genetic variation within a clinical study population correlates with response to the treatment(s) under evaluation. If genetic variation is found to predict efficacy or adverse events, the data might inform optimal use of therapies in the patient population. Furthermore, it is important to evaluate germline DNA variation across the genome in order to interpret tumor-specific DNA mutations.
The Sponsor will collect tissue from archival samples, pre-treatment sample, and post-treatment sample. The Sponsor will get blood samples every 6 weeks during treatment period. This research will include exome sequencing, RNA sequencing, and immunohistochemistry.
Comprehensive genomic profiling & immune biomarker exploration will be performed for all samples to find the predictive biomarkers for combination therapy of olaparib, bevacizumab, and pembrolizumab.

1) DNA/ RNA analyses from tumor/blood
Tumor and blood samples from this study will be examined to identify BRCA reversion mutation and HRD status changes. The application of next generation sequencing has provided scientists the opportunity to identify tumor-specific DNA changes. RNA sequencing will be performed to identify immune dynamic changes between pre-treatment and progression
2) Analysis of immune marker using FACS from tumor/blood

We will Examine the phenotypic characteristics of CD4 and CD8 T cells in terms of various immune check points (PD-1, CTLA4, TIM3, LAG3) and memory markers (CD45RO+CCR7+) in fresh tissue and PBMC by FACS on pre-treatment & at progression.
We will examine the tumor infiltrating immune cells including Treg cells and MDSCs on pre-treatment & at progression.
3) PD-L1 testing and multiplex IHC from tumor/blood
Tumor samples from this study may undergo protemic analyses (eg. PD-L1 IHC). PD-L1 expression level at pre-treatment and at progression will be checked. Using established platform of Vectra, multispectral imaging will be performed to identify tumor microenvironment comprehensively.
ctDNA, PBMCs and plasma will also be isolated and stored for exploratory biomarker analysis.

5.0 METHODOLOGY

5.1 Study Population

Female participants of at least 20 years of age with EOC, fallopian tube cancer, or primary peritoneal cancer will be enrolled in this study.

5.1.1 Participant Inclusion Criteria

Participants are eligible to be included in the study only if all of the following criteria apply:

Type of Participant and Disease Characteristics

1. Participant has histologically confirmed diagnosis of high-grade predominantly serous, endometrioid, carcinosarcoma, mixed mullerian with high-grade serous component, clear cell, or low-grade serous OC, primary peritoneal cancer, or fallopian tube cancer will be enrolled in this study (only up to 8 patients with clear cell carcinoma will be included and mucinous carcinoma will not be included).
2. Participant has received 2 previous courses of platinum-containing therapy, and has disease that was considered platinum sensitive following the penultimate (next to last) platinum course (more than 6 months' period between penultimate platinum regimen and progression of disease)
3. Participant has responded to last the platinum regimen (complete or partial response), remains in response and is enrolled on study within 8 weeks of completion of the last platinum regimen
4. Participant is able to provide tumor slides for prospective testing of BRCA 1/2 and PD-L1 status prior to enrollment

Demographics

5. Female participants who are at least 20 years of age on the day of signing informed consent with

6. Participant has an Eastern Cooperative Oncology Group (ECOG) performance status of 0 or 1, as assessed within 7 days prior to enrollment.

Female participants:

7. A female participant is eligible to participate if she is not pregnant (see Appendix 3), not breastfeeding, and at least one of the following conditions applies:
   a.) Not a woman of childbearing potential (WOCBP) as defined in Appendix 3
   OR
   b.) A WOCBP who agrees to follow the contraceptive guidance in Appendix 3 during the treatment period and for at least 120 days following the last dose of pembrolizumab and olaparib and at least 210 days following the last dose of chemotherapy or bevacizumab.

Informed Consent

8. The participant (or legally acceptable representative if applicable) provides written informed consent for the trial. The participant may also provide consent for future biomedical research; however, the participant may participate in the main study without participating in future biomedical research.

Laboratory Values

9. Participant has adequate organ function as defined in the following table (Table 1).; all screening laboratory tests should be performed within 10 days prior to the start of study treatment.

Table 1 Adequate Organ Function Laboratory Values

System	Laboratory Value
Hematological	
Absolute neutrophil count (ANC)	≥1500/µL
Platelets	≥100 000/µL
Hemoglobin	≥9.0 g/dL or ≥5.6 mmol/L[a]
Renal	
Creatinine OR<br>Measured or calculated[b] creatinine clearance (GFR can also be used in place of creatinine or CrCl)	≤1.5 × ULN OR<br>≥30 mL/min for participant with creatinine levels >1.5 × institutional ULN
Hepatic	
Total bilirubin	≤1.5 ×ULN OR direct bilirubin ≤ULN for participants with total bilirubin levels >1.5 × ULN

AST (SGOT) and ALT (SGPT)	≤2.5 × ULN (≤5 × ULN for participants with liver metastases)
Coagulation	
International normalized ratio (INR) OR prothrombin time (PT)<br>Activated partial thromboplastin time (aPTT)	≤1.5 × ULN unless participant is receiving anticoagulant therapy as long as PT or aPTT is within therapeutic range of intended use of anticoagulants
ALT (SGPT)=alanine aminotransferase (serum glutamic pyruvic transaminase); AST (SGOT)=aspartate aminotransferase (serum glutamic oxaloacetic transaminase); GFR=glomerular filtration rate; ULN=upper limit of normal.<br>[a] Criteria must be met without erythropoietin dependency.<br>[b] Creatinine clearance (CrCl) should be calculated per institutional standard.<br>Note: This table includes eligibility-defining laboratory value requirements for treatment; laboratory value requirements should be adapted according to local regulations and guidelines for the administration of specific chemotherapies.	

5.1.2 Participant Exclusion Criteria

Participants are excluded from the study if any of the following criteria apply:

Medical Conditions

1. Participant has mucinous, germ cell, or borderline tumor of the ovary.
2. Participant has a known or suspected deleterious mutation (germline or somatic) in either BRCA1 or BRCA2
3. Participant has a history of non-infectious pneumonitis that required treatment with steroids or currently has pneumonitis
4. Participant either has myelodysplastic syndrome (MDS)/ acute myeloid leukemia (AML) or has features suggestive of MDS/AML.
5. Participant has a known additional malignancy that is progressing or has required active treatment within the past *3* years.

   Note: Participants with basal cell carcinoma of the skin, squamous cell carcinoma of the skin, or carcinoma in situ (e.g. breast carcinoma, cervical cancer in situ, endometrial carinoma) that have undergone potentially curative therapy are not excluded.

   Note: Participants with synchronous primary endometrial cancer or a past history of primary endometrial cancer that met the following conditions are not excluded: Stage not greater than IA: no more than superficial myometrial invasion.

6. Participant has known active CNS metastases and/or carcinomatous meningitis. Participants with previously treated brain metastases may participate provided they are radiologically stable, i.e. without evidence of progression for at least 4 weeks by repeat

imaging (note that the repeat imaging should be performed during study screening), clinically stable and without requirement of steroid treatment for at least 14 days prior to first dose of study treatment.

7. Participant has a diagnosis of immunodeficiency or is receiving chronic systemic steroid therapy (in dosing exceeding 10 mg daily of prednisone equivalent) or any other form of immunosuppressive therapy within 7 days prior to the first dose of study drug.

8. Participant has active autoimmune disease that has required systemic treatment in the past 2 years (i.e. with use of disease modifying agents, corticosteroids or immunosuppressive drugs). Replacement therapy (eg., thyroxine, insulin, or physiologic corticosteroid replacement therapy for adrenal or pituitary insufficiency, etc.) is not considered a form of systemic treatment.

9. Participant has a known history of active TB (Bacillus Tuberculosis).
10. Participant has an active infection requiring systemic therapy.

11. Participant has a history or current evidence of any condition, therapy, or laboratory abnormality that might confound the results of the study, interfere with the subject's participation for the full duration of the study, or is not in the best interest of the subject to participate, in the opinion of the treating investigator.

12. Participant has had underwent chemotherapy to treat borderline tumor.
13. Participant has known psychiatric or substance abuse disorders that would interfere with cooperation with the requirements of the trial.

14. Participant has a known history of Human Immunodeficiency Virus (HIV).

15. Participant has a known history of Hepatitis B (defined as Hepatitis B surface antigen [HBsAg] reactive) or known active Hepatitis C virus (defined as HCV RNA [qualitative] is detected) infection. Note: no testing for Hepatitis B and Hepatitis C is required unless mandated by local health authority.

16. Participant is either unable to swallow orally administered medication or has a gastrointestinal disorder affecting absorption (eg. Gasterectomy, partial bowel obstruction, malabsorption)
17. Participant has uncontrolled hypertension, defined as systolic > 140 mmHg or diastolic > 90 mmHg documented by 2 blood pressure readings taken at least 1 hour apart.

    Note: Use of antihypertensive medications to control blood pressure is allowed.

18. Participant has current, clinically relevant bowel obstruction, abdominal fistula or gastrointestinal perforation, related to underlying EOC.
19. Participant has a history of hemorrhage, hemoptysis or active gastrointestinal bleeding within 6 months prior to randomization.

20. WOCBP who has a positive urine pregnancy test within 72 hours prior to allocation (see Appendix 3). If the urine test is positive or cannot be confirmed as negative, a serum pregnancy test will be required.

Note: in the event that 72 hours have elapsed between the screening pregnancy test and the first dose of study treatment, another pregnancy test (urine or serum) must be performed and must be negative in order for subject to start receiving study medication.

Prior/ Concomitant Therapy

21. Participant has received prior therapy with an anti-PD-1, anti-PD-L1, or anti-PD-L2 agent or with an agent directed to another stimulatory or co-inhibitory T-cell receptor (eg, CTLA-4, OX-40, CD137).

Note: Participant who received prior therapy with either olaparib or any other PARP inhibitor is allowed. Participant who received prior therapy with bevacizumab or anti-angiogenic agent is allowed.

22. Participant has received a live vaccine within 30 days prior to the first dose of study drug. Examples of live vaccines include, but are not limited to, the following: measles, mumps, rubella, varicella/zoster (chicken pox), yellow fever, rabies, Bacillus Calmette–Guérin (BCG), and typhoid vaccine. Seasonal influenza vaccines for injection are generally killed virus vaccines and are allowed; however, intranasal influenza vaccines (eg, FluMist®) are live attenuated vaccines and are not allowed.
23. Participant has severe hypersensitivity (≥Grade 3) to pembrolizumab, olaparib, or bevacizumab and/or any of its excipients.

24. Participant is currently receiving either strong (eg. Itraconzaole, telithromycin, clarithromycin, protease inhibitors boosted with ritonavir or cobicistat, indinavir, saquinavir, nelfinavir, boceprevir, telaprevir) or moderate (eg. Ciprofloxacin, erythromycin, diltiazem, fluconazole, verapamil) inhibitors of cytochrome P450 (CYP)3A4 that cannot be discontinued prior to starting olaparib and for the duration of the study. The required washout period prior to starting olaparib is 2 weeks.

    Note: A current list of strong/moderate inhibitors of CYP3A4 can be found at the following website:
    https://www.fda.gov/Drugs/DevelopmentApprovalProcess/DevelopmentResources/DrugInteractionsLabeling
25. Participant is currently receiving either strong (eg. Phenobarbital, enzalutamide, phenytoin, rifampin, rifabutin, rifapentine, carbamazepine, nevirapine, and St John's' Wort) or moderate (eg. Bosentan, efavirenz, modafinil) inducers of cytochrome P450 (CYP)3A4 that cannot be discontinued prior to starting olaparib and for the duration of the study. The required washout period prior to starting olaparib is 5 weeks for phenobarbital and 3 weeks for other agents.

    Note: A current list of strong/moderate inducers of CYP3A4 can be found at the following

website:
https://www.fda.gov/Drugs/DevelopmentApprovalProcess/DevelopmentResources/DrugInteractionsLabeling

Prior/Concurrent Clinical Study Experience

26. Participant is currently participating in or has participated in a study of an investigational agent or has used an investigational device within 4 weeks prior to the first dose of study treatment.
    Note: Participants who have entered the follow-up phase of an investigational study may participate as long as it has been 4 weeks after the last dose of the previous investigational agent.

Diagnostic Assessments

27. Participant has resting electrocardiogram (ECG) indicating uncontrolled, potentially reversible cardiac conditions, as judged by the investigator (eg. Unstable ischemia, uncontrolled symptomatic arrhythmia, congestive heart failure, QTcF prolongation > 500 ms, electrolyte disturbances, etc.), or participant has congenital long QT syndrome.

Other Exclusion

28. Participant has received prior systemic anti-cancer therapy including investigational agents within 4 weeks prior to allocation. Participant either had major surgery within 2 weeks of enrollment or has not recovered from any effects of any major surgery.

    Note: Participants must have recovered from all AEs due to previous therapies to ≤Grade 1 or baseline. Participants with ≤Grade 2 neuropathy may be eligible.

    Note: If participant received major surgery, they must have recovered adequately from the toxicity and/or complications from the intervention prior to starting study treatment.

29. Participant has received prior radiotherapy within 2 weeks of start of study treatment. Participants must have recovered from all radiation-related toxicities, not require corticosteroids, and not have had radiation pneumonitis. A 1-week washout is permitted for palliative radiation (≤2 weeks of radiotherapy) to non-CNS disease.

30. Participant has had allogenic tissue/solid organ transplant, has received previous allogenic bone-marrow transplant, or has received double umbilical cord transplantation.

31. Participant, in the judgement of the investigator, is unlikely to comply with the study procedures, restrictions, and requirements of the study.

5.1.3 Lifestyle Restrictions

5.1.3.1 Meals and Dietary Restrictions

During the treatment period, participants should avoid grapefruit, grapefruit juice, Seville oranges, Seville orange juice, and St. John's Wort (tablet or tea) while receiving study treatment. Otherwise, participants should maintain a normal diet unless modifications are required to manage an AE such as diarrhea, nausea or vomiting.

5.1.3.2 Contraception

The study treatment may have adverse effects on a fetus in utero. Refer to Appendix 3 for approved methods of contraception.

5.1.4 Pregnancy

If a participant inadvertently becomes pregnant while on treatment with pembrolizumab, the participant will be immediately discontinued from study treatment. The site will contact the participant at least monthly and document the participant's status until the pregnancy has been completed or terminated. The outcome of the pregnancy will be reported to Merck within 2 working days if the outcome is a serious adverse experience (eg, death, abortion, congenital anomaly, or other disabling or life-threatening complication to the mother or newborn). The study Investigator will make every effort to obtain permission to follow the outcome of the pregnancy and report the condition of the fetus or newborn to Merck.

5.1.5 Use in Nursing Women

It is unknown whether pembrolizumab is excreted in human milk. Since many drugs are excreted in human milk, and because of the potential for serious adverse reactions in the nursing infant, participants who are breast-feeding are not eligible for enrollment.

5.2 Trial Treatments

The treatment to be used in this trial is outlined below in Table 2

Table 2 Trial Treatment

Drug	Dose/Potency	Dose Frequency	Route of Administration	Regimen/Treatment Period	Use
Pembrolizumab	200 mg	Q3W	IV infusion	Q3W ; Day 1 of each 3 week cycle from cycle 2	Experimental
Olaparib	300mg	BID	Oral	during each treatment cycle	Experimental
Bevacizumab	15mg/kg or 7.5mg/kg	Q3W	IV infusion	Q3W; Day 1 of each 3 week cycle	Background therapy
Abbreviations: BID = twice daily; Q3W = every 3 weeks					

Trial treatment should begin on the day of allocation or as close as possible to the date on which treatment is allocated/assigned.

5.2.1 Timing of Dose Administration

Trial treatment should be administered on Day 1 of each cycle after all procedures/assessments have been completed as detailed on the Trial Flow Chart (Section 6.0). Trial treatment may be administered up to 3 days before or after the scheduled Day 1 of each cycle due to administrative reasons.

All trial treatments will be administered on an outpatient basis.

Pembrolizumab 200 mg will be administered as a 30 minute IV infusion every 3 weeks from cycle 2. Sites should make every effort to target infusion timing to be as close to 30 minutes as possible. However, given the variability of infusion pumps from site to site, a window of -5 minutes and +10 minutes is permitted (i.e., infusion time is 30 minutes: -5 min/+10 min).

The Pharmacy Manual contains specific instructions for the preparation of the pembrolizumab infusion fluid and administration of infusion solution.

5.2.2 Dose Modification and toxicity management for immune-related AEs associated with pembrolizumab

AEs associated with pembrolizumab exposure may represent an immunologic etiology. These immune-related AEs (irAEs) may occur shortly after the first dose or several months after the last dose of pembrolizumab treatment and may affect more than on body system simultaneously. Therefore, early recognition and initiation of treatment is critical to reduce complications. Based on existing clinical study data, most irAEs were reversible and could be managed with interruptions of pembrolizumab, administration of corticosteroids and/or other supportive care. For suspected irAEs, ensure adequate evaluation to confirm etiology or exclude other causes. Additional procedures or tests such as bronchoscopy, endoscopy, skin biopsy may be included as part of the evaluation. Based on the severity of irAEs, withhold or permanently discontinue pembrolizumab and administer corticosteroids. Dose modification and toxicity management guidelines for irAEs associated with pembrolizumab are provided in Table 3.

Table 3 Dose modification and toxicity management guidelines for immune-related AEs associated with pembrolizumab

General instructions:

1. Severe and life-threatening irAEs should be treated with IV corticosteroids followed by oral steroids. Other immunosuppressive treatment should begin if the irAEs are not controlled by corticosteroids.
2. Pembrolizumab must be permanently discontinued if the irAE does not resolve or the corticosteroid dose is not ≤10 mg/day within 12 weeks of the last pembrolizumab treatment.
3. The corticosteroid taper should begin when the irAE is ≤ Grade 1 and continue at least 4 weeks.
4. If pembrolizumab has been withheld, pembrolizumab may resume after the irAE decreased to ≤ Grade 1 after corticosteroid taper.

irAEs	Toxicity grade (CTCAE V5.0)	Action with pembrolizumab	Corticosteroid and/or other therapies	Monitoring and follow-up
Pneumonitis	Grade 2	Withhold	• Administer corticosteroids (initial dose of 1 - 2 mg/kg prednisone or equivalent) followed by taper<br>• Add prophylactic antibiotics for opportunistic infections	• Monitor participants for signs and symptoms of pneumonitis<br>• Evaluate participants with suspected pneumonitis with radiographic imaging and initiate corticosteroid treatment
	Grade 3 or 4, or recurrent Grade 2	Permanently discontinue		
Diarrhea / Colitis	Grade 2 or 3	Withhold		• Monitor participants for signs and symptoms of enterocolitis (ie, diarrhea, abdominal pain, blood or mucus in stool with or without fever) and of bowel perforation (ie, peritoneal signs and ileus)

	Grade 4 or recurrent Grade 3	Permanently discontinue	• Administer corticosteroids (initial dose of 1 - 2 mg/kg prednisone or equivalent) followed by taper	• Participants with ≥Grade 2 diarrhea suspecting colitis should consider GI consultation and performing endoscopy to rule out colitis<br>• Participants with diarrhea/colitis should be advised to drink liberal quantities of clear fluids. If sufficient oral fluid intake is not feasible, fluid and electrolytes should be substituted via IV infusion
AST or ALT elevation or Increased Bilirubin	Grade 2 [a]	Withhold	• Administer corticosteroids (initial dose of 0.5 - 1 mg/kg prednisone or equivalent) followed by taper	• Monitor with liver function tests (consider weekly or more frequently until liver enzyme value returned to baseline or is stable)
	Grade 3 [b] or 4 [c]	Permanently discontinue	• Administer corticosteroids (initial dose of 1 - 2 mg/kg prednisone or equivalent) followed by taper	
Type 1 diabetes mellitus (T1DM) or Hyperglycemia	New onset T1DM or Grade 3 or 4 hyperglycemia associated with evidence of β-cell failure	Withhold [d]	• Initiate insulin replacement therapy for participants with T1DM<br>• Administer anti-hyperglycemic in participants with hyperglycemia	• Monitor participants for hyperglycemia or other signs and symptoms of diabetes
Hypophysitis	Grade 2	Withhold		• Monitor for signs and symptoms of hypophysitis (including hypopituitarism and adrenal insufficiency)

	Grade 3 or 4	Withhold or permanently discontinue [d]	• Administer corticosteroids and initiate hormonal replacements as clinically indicated	
Hyperthyroidism	Grade 2	Continue	• Treat with non-selective beta-blockers (eg, propranolol) or thionamides as appropriate	• Monitor for signs and symptoms of thyroid disorders
	Grade 3 or 4	Withhold or permanently discontinue [d]		
Hypothyroidism	Grade 2, 3, or 4	Continue	• Initiate thyroid replacement hormones (eg, levothyroxine or liothyronine) per standard of care	• Monitor for signs and symptoms of thyroid disorders
Nephritis and renal dysfunction: grading according to increased creatinine or acute kidney injury	Grade 2	Withhold	• Administer corticosteroids (prednisone 1 – 2 mg/kg or equivalent) followed by taper	• Monitor changes of renal function
	Grade 3 or 4	Permanently discontinue		
Myocarditis	Grade 1 or 2	Withhold	• Based on severity of AE administer corticosteroids	• Ensure adequate evaluation to confirm etiology and/or exclude other causes
	Grade 3 or 4	Permanently discontinue		
All Other immune-related AEs	Intolerable/persistent Grade 2	Withhold	• Based on severity of AE administer corticosteroids	• Ensure adequate evaluation to confirm etiology or exclude other causes
	Grade 3	Withhold or discontinue		

		based on the event [e].		
	Grade 4 or recurrent Grade 3	Permanently discontinue		

[a] AST/ALT: >3.0 - 5.0 x ULN if baseline normal; >3.0 - 5.0 x baseline, if baseline abnormal; bilirubin:>1.5 - 3.0 x ULN if baseline normal; >1.5 - 3.0 x baseline if baseline abnormal

[b] AST/ALT: >5.0 to 20.0 x ULN, if baseline normal; >5.0 - 20.0 x baseline, if baseline abnormal; bilirubin:>3.0 - 10.0 x ULN if baseline normal; >3.0 - 10.0 x baseline if baseline abnormal

[c] AST/ALT: >20.0 x ULN, if baseline normal; >20.0 x baseline, if baseline abnormal; bilirubin: >10.0 x ULN if baseline normal; >10.0 x baseline if baseline abnormal

[d] The decision to withhold or permanently discontinue pembrolizumab is at the discretion of the investigator or treating physician. For participants with Grade 3 or 4 immune-related endocrinopathy where withhold of pembrolizumab is required, pembrolizumab may be resumed when AE resolves to ≤ Grade 2 and is controlled with hormonal replacement therapy or achieved metabolic control (in case of T1DM)

[e] Events that require discontinuation include but are not limited to: Guillain-Barre Syndrome, encephalitis, Stevens-Johnson Syndrome and toxic epidermal necrolysis.

<u>Dose modification and toxicity management of infusion-reactions related to pembrolizumab</u>

Pembrolizumab may cause severe or life threatening infusion-reactions including severe hypersensitivity or anaphylaxis. Signs and symptoms usually develop during or shortly after drug infusion and generally resolve completely within 24 hours of completion of infusion. Dose modification and toxicity management guidelines on pembrolizumab associated infusion reaction are provided in Table 4.

Table 4 Pembrolizumab Infusion Reaction Dose modification and Treatment Guidelines

NCI CTCAE Grade	Treatment	Premedication at Subsequent Dosing
**Grade 1**<br>Mild reaction; infusion interruption not indicated; intervention not indicated	Increase monitoring of vital signs as medically indicated until the participant is deemed medically stable in the opinion of the investigator.	None
**Grade 2**<br>Requires therapy or infusion interruption but responds promptly to symptomatic treatment (e.g., antihistamines, NSAIDs, narcotics, IV fluids); prophylactic medications indicated for ≤24 hrs	**Stop Infusion.**<br>Additional appropriate medical therapy may include but is not limited to:<br>IV fluids<br>Antihistamines<br>NSAIDs<br>Acetaminophen<br>Narcotics<br>Increase monitoring of vital signs as medically indicated until the participant is deemed medically stable in the opinion of the investigator.<br>If symptoms resolve within 1 hour of stopping drug infusion, the infusion may be restarted at 50% of the original infusion rate (e.g. from 100 mL/hr to 50 mL/hr). Otherwise dosing will be held until symptoms resolve and the participant should be premedicated for the next scheduled dose.<br>**Participants who develop Grade 2 toxicity despite adequate premedication should be permanently discontinued from further study drug treatment**	Participant may be premedicated 1.5h (± 30 minutes) prior to infusion of _____ with:<br>Diphenhydramine 50 mg po (or equivalent dose of antihistamine).<br>Acetaminophen 500-1000 mg po (or equivalent dose of analgesic).

**Grades 3 or 4**<br>Grade 3:<br>Prolonged (i.e., not rapidly responsive to symptomatic medication and/or brief interruption of infusion); recurrence of symptoms following initial improvement; hospitalization indicated for other clinical sequelae (e.g., renal impairment, pulmonary infiltrates)<br>Grade 4:<br>Life-threatening; pressor or ventilatory support indicated	**Stop Infusion.**<br>Additional appropriate medical therapy may include but is not limited to:<br>Epinephrine**<br>IV fluids<br>Antihistamines<br>NSAIDs<br>Acetaminophen<br>Narcotics<br>Oxygen<br>Pressors<br>Corticosteroids<br>Increase monitoring of vital signs as medically indicated until the participant is deemed medically stable in the opinion of the investigator.<br>Hospitalization may be indicated.<br>**In cases of anaphylaxis, epinephrine should be used immediately.<br>**Participant is permanently discontinued from further study drug treatment.**	No subsequent dosing
Appropriate resuscitation equipment should be available at the bedside and a physician readily available during the period of drug administration.<br>For further information, please refer to the Common Terminology Criteria for Adverse Events v5.0 (CTCAE) at http://ctep.cancer.gov		

<u>Other allowed dose interruption for pembrolizumab</u>

Pembrolizumab may be interrupted for situations other than treatment-related AEs such as medical / surgical events or logistical reasons not related to study therapy. Participants should be placed back on study therapy within 3 weeks of the scheduled interruption, unless otherwise discussed with the Sponsor. The reason for interruption should be documented in the patient's study record.

5.2.3 Olaparib Dosing Modifications

The dose of olaparib can be reduced to 250 mg BID initially and then to 200 mg BID as needed. If the 200 mg BID dose is not tolerable, no further dose reduction is allowed and study treatment should be discontinued. Once dose is reduced, escalation is not permitted (except following concomitant treatment with CYP3A4 inhibitors (Table 8).
The reason for the dose interruption or reduction should be captured on the appropriate eCRF. If either olaparib is discontinued during the maintenance phase due to toxicity, the participant may continue pembrolizumab if the criteria outlined in Section 5.2.2 have not been met.

5.2.3.1 Management of Hematologic Toxicity

Any hematological toxicity observed during the study could be managed by a brief interruption of study treatment or a dose reduction of olaparib (Table 5 and Table 6). Repeated interruptions, not exceeding 4 weeks duration, are allowed as required. If the interruption is any longer, the study team must be informed.

Table 5 Management of Anemia

Toxicity	NCI CTCAE Grade	Action Taken
Hemoglobin (Hb)	Grade 2<br><br>(10 but ≥ 8 g/dL)	**First Occurrence:**<br>Give appropriate supportive treatment and investigate causality.<br>• Investigator judgement to either continue olaparib with supportive treatment (eg, transfusion) or interrupt olaparib dosing for a maximum of 4 weeks. Treatment can be restarted if Hb has recovered to >9 g/dL.<br>**Subsequent Recurrence:**<br>• **Hb <9 but ≥8 g/dL**: Interrupt olaparib for a maximum of 4 weeks until Hb improves to >9 g/dL. Upon recovery, reduce the dose of olaparib to 250 mg/matching tablet BID. A second dose reduction to 200 mg/matching tablet BID
	Grade 3	Give appropriate supportive treatment (eg, transfusion) and investigate causality.<br>• Interrupt olaparib, for a maximum of 4 weeks, until Hb improves to ≥9 g/dL.

	(<8 g/dL)	• Upon recovery, reduce the dose of olaparib to 250 mg/matching tablet BID. A second dose reduction to 200mg/matching tablet BID may be considered if additional decreases in Hb occur.
Abbreviations: BID = twice daily; CTCAE = Common Terminology Criteria for Adverse Events; Hb = hemoglobin; NCI = National Cancer Institute.<br>Note: Common treatable causes of anemia (eg, iron, vitamin B12 or folate deficiencies and hypothyroidism) should be investigated and appropriately managed. In some cases management of anemia may require blood transfusions. The management of prolonged hematological toxicities is detailed in Section 5.2.3.2		

Table 6 Management of Neutropenia, Leukopenia, and Thrombocytopenia

Toxicity	NCI CTCAE Grade	Action Taken
Neutropenia, Leukopenia, or Thrombocytopenia	Grade 1 or 2	Investigator judgement to either continue olaparib or interrupt dosing for a maximum of 4 weeks. Give appropriate supportive treatment and investigate causality.
	Grade 3 or 4	• Interrupt olaparib, for a maximum of 4 weeks, until event recovers to ≤Grade 1.<br>• Repeated incidence: reduce the dose of olaparib to 250 mg/matching tablet BID. A second dose reduction to 200 mg/matching tablet BID may be considered if additional Grade 3 or 4 events occur.
Abbreviations: BID = twice daily; CTCAE = Common Terminology Criteria for Adverse Events; G-CSF = granulocyte colony-stimulating factor; NCI = National Cancer Institute.<br>• AEs of neutropenia and leukopenia should be managed as deemed appropriate by the investigator with close follow-up and interruption of study treatment if CTCAE Grade 3 or worse neutropenia occurs.<br>• Primary prophylaxis with G-CSF is not recommended; however, if a participant develops febrile neutropenia, study treatment should be stopped and appropriate management including G-CSF should be given according to local hospital guidelines. Please note that G-CSF should not be used within at least 24 hours (7 days for pegylated G-CSF) of the last dose of study treatment unless absolutely necessary.<br>• Platelet transfusions, if indicated, should be done according to local hospital guidelines.<br>• The management of prolonged hematological toxicities is detailed in Section 5.2.3.2		

5.2.3.2 Management of Prolonged Hematologic Toxicities

If a participant develops prolonged hematological toxicity such as:
• ≥2-week interruption/delay in olaparib due to NCI CTCAE Grade 3 or worse anemia and/or the development of blood transfusion dependence
• ≥2-week interruption/delay in olaparib due to NCI CTCAE Grade 3 or worse neutropenia (absolute neutrophil count <1 × 10^9/L)
• ≥2-week interruption/delay in olaparib due to NCI CTCAE Grade 3 or worse thrombocytopenia and/or development of platelet transfusion dependence (platelets <50 × 10^9/L)
Differential blood count, including reticulocytes and peripheral blood smear, should be checked weekly. If any blood parameters remain clinically abnormal after the dosing of

olaparib has been interrupted for 4 weeks, the participant should be referred to hematologist for further investigations. Bone marrow analysis and/or blood cytogenetic analysis should be considered, according to local regulation and/or standard institutional hematological practice. Olaparib should be discontinued if blood counts do not recover to NCI CTCAE Grade 1 or better within 4 weeks of dose interruption. Development of confirmed MDS or other clonal blood disorder should be reported as an SAE and full reports must be provided by the investigator to the Sponsor as outlined in Section 7.2. Olaparib treatment should be discontinued for confirmed MDS and/or AML.

5.2.3.3 Management of Non-hematological Toxicity

Repeat dose interruptions are allowed as required, for a maximum of 4 weeks on each occasion. If the interruption is longer 4 weeks, the Sponsor must be informed. Where toxicity reoccurs following re-challenge with olaparib, and where further dose interruptions are considered inadequate for management of toxicity, either a dose reduction should be considered (Section 5.2.3.1) or the participant must permanently discontinue study treatment. Treatment must be interrupted if any NCI CTCAE Grade 3 or 4 AE occurs that the investigator considers to be related to administration of olaparib.

5.2.3.3.1 Management of New or Worsening Pulmonary Symptoms

If new or worsening pulmonary symptoms (eg, dyspnea) or radiological abnormalities occur in the absence of a clear diagnosis, study treatment must be interrupted (Section 6.6.3) and further diagnostic workup (including a high resolution computed tomography [CT] scan) should be performed to exclude pneumonitis.
Following investigation, if no evidence of abnormality is observed on CT imaging and symptoms resolve, then olaparib may be restarted, if deemed appropriate by the investigator. If significant pulmonary abnormalities are identified, these need to be discussed with the Clinical Director.

5.2.3.3.2 Management of Nausea and Vomiting

Events of nausea and vomiting are known to be associated with olaparib treatment. These events are generally mild to moderate (NCI CTCAE Grade 1 or 2) in severity, intermittent, and manageable on continued treatment. The first onset generally occurs in the first month of treatment for nausea and within the first 6 months of treatment for vomiting. For nausea, the incidence generally plateaus at around 9 months, and for vomiting at around 6 to 7 months. No routine prophylactic antiemetic treatment is required at the start of dosing with olaparib; however, participants should receive appropriate antiemetic treatment at the first onset of nausea or vomiting and as required thereafter, in accordance with local regulations or institutional guidelines. Alternatively, olaparib tablets can be taken with a light meal/snack (eg, 2 pieces of toast or a couple of biscuits). As per international guidance on antiemetic use in cancer patients (European Society for Medical Oncology, National Comprehensive Cancer Network), generally a single agent antiemetic should be considered (eg, dopamine receptor antagonist, antihistamines, or dexamethasone).

5.2.3.3.3 Management of Renal Impairment

If subsequent to study entry and while still on study therapy, a participant's estimated creatinine clearance (CrCl) falls below the threshold for study inclusion (≥51 mL/min), retesting should be performed promptly.
A dose reduction is recommended for participants who develop moderate renal impairment (calculated CrCl between 31 and 50 mL/min as calculated by either Cockcroft-Gault equation or based on a 24-hour urine test) for any reason during the course of the study (Table 7).

Table 7 Dose Reduction of Olaparib to Manage Moderate Renal Impairment

Initial Dose	Moderate Renal Impairment[a]
300 mg BID	200 mg BID
Abbreviation: BID = twice daily<br>a. Creatinine clearance of 30 to 50 mL/min as calculated by either Cockcroft-Gault equation of based on 24-hour urine test.	

Because the CrCl determination is only an estimate of renal function, in instances where the CrCl falls to between 31 and 50 mL/min, the investigator should use his or her discretion in determining whether a dose change or discontinuation of therapy is warranted. Olaparib has not been studied in participants with severe renal impairment (CrCl≤30 mL/min) or end-stage renal disease; if participants develop severe impairment or end stage disease, it is recommended that olaparib be discontinued.

5.2.3.4 Interruption for Intercurrent Non-Toxicity-Related Event(12.1.5.4)

Olaparib dose interruption for conditions other than toxicity resolution should be kept as short as possible. If a participant cannot restart study treatment within 4 weeks for resolution of intercurrent conditions not related to disease progression or toxicity, the case should be discussed with the Clinical Director. All dose reductions and interruptions (including any missed doses), and the reasons for the reductions/interruptions are to be recorded in the eCRF. Olaparib should be stopped at least 3 days prior to planned surgery and can be restarted when the wound has healed. It is not required to stop olaparib for any needle biopsy procedure.
Olaparib should be discontinued for a minimum of 3 days before a participant undergoes radiation treatment and should be restarted within 4 weeks as long as any bone marrow toxicity has recovered.
Because the AEs related to olaparib may include asthenia, fatigue and dizziness, participants should be advised to use caution while driving or using machinery if these symptoms occur.

5.2.3.5 Dose Reductions for Concurrent CYP3A4 Inhibitor Use((12.1.5.5)

Strong or moderate CYP3A inhibitors should not be taken with olaparib. If, at any time after

starting olaparib in the Maintenance Period, there is no suitable alternative concomitant medication then the dose of olaparib should be reduced for the period of concomitant administration as described in Table 8. After the washout of the inhibitor is complete (outlined in Section 5.1.2), the olaparib dose can be reescalated. The dose reduction of olaparib should be recorded in the eCRF with the reason documented as concomitant CYP3A inhibitor use.

Table 8 Dose Reduction of Olaparib With a Strong or Moderate CYP3A4 Inhibitor

Initial Dose	Strong CYP3A Inhibitor	Moderate CYP3A Inhibitor
300mg BID	100 mg BID	150 mg BID
Abbreviation: BID = twice daily; CYP = Cytochrome P450		

5.2.4 Management of Overlapping Toxicities((12.1.6)

Both olaparib and pembrolizumab treatment may be associated with the development of pneumonitis. Treatment with olaparib must be held for any grade of pneumonitis. Treatment with pembrolizumab must be held for pneumonitis ≥Grade 2 (Table 3).
When the pneumonitis resolves to <Grade 2, then pembrolizumab may be resumed as per guidelines in Table 3. Olaparib may be restarted once pneumonitis has completely resolved. Study treatment must be discontinued for recurrent Grade 2 pneumonitis (Section 7.1.4).

5.2.5 Bevacizumab Dose Modification

Bevacizumab, may be reduced, interrupted, or discontinued at the investigator’s discretion per the approved product label and local regulations. If bevacizumab is interrupted or discontinued, pembrolizumab and olaparib may be continued.

5.3 Randomization or Treatment Allocation

Not available

5.4 Stratification

Not available

5.5 Concomitant Medications/Vaccinations (allowed & prohibited)

Medications or vaccinations specifically prohibited in the exclusion criteria are not allowed during the ongoing trial. If there is a clinical indication for one of these or other medications or vaccinations specifically prohibited during the trial, discontinuation from trial therapy or vaccination may be required. The final decision on any supportive therapy or vaccination rests with the investigator and/or the participants's primary physician.

5.5.1 Acceptable Concomitant Medications(12.2.7.1)

All treatments that the investigator considers necessary for a participant's welfare may be administered at the discretion of the investigator in keeping with the community standards of medical care. All concomitant medication will be recorded on the case report form (CRF) including all prescription, over-the-counter (OTC), herbal supplements, and IV medications and fluids. If changes occur during the trial period, documentation of drug dosage, frequency, route, and date may also be included on the CRF.

All concomitant medications received within 28 days before the first dose of trial treatment and 30 days after the last dose of trial treatment should be recorded. Concomitant medications administered after 30 days after the last dose of trial treatment should be recorded for SAEs and ECIs as defined in Section 7.2.

5.5.2 Prohibited Concomitant Medications

Parcticipants are prohibited from receiving the following therapies during the Screening and Treatment Phase (including retreatment for post-complete response relapse) of this trial:

- Antineoplastic systemic chemotherapy or biological therapy
- Immunotherapy not specified in this protocol
- Chemotherapy not specified in this protocol
- Investigational agents other than pembrolizumab
- Radiation therapy
    - Note: Radiation therapy to a symptomatic solitary lesion or to the brain may be allowed at the investigator's discretion.
- Live vaccines within 30 days prior to the first dose of study treatment and while participating in the study. Examples of live vaccines include, but are not limited to, the following: measles, mumps, rubella, varicella/zoster, yellow fever, rabies, BCG, and typhoid vaccine. Seasonal influenza vaccines for injection are generally killed virus vaccines and are allowed; however, intranasal influenza vaccines (eg, FluMist®) are live attenuated vaccines and are not allowed.
- Systemic glucocorticoids for any purpose other than to modulate symptoms from an event of clinical interest of suspected immunologic etiology. The use of physiologic doses of corticosteroids may be approved after consultation with the Sponsor.

Participants who, in the assessment by the investigator, require the use of any of the aforementioned treatments for clinical management should be removed from the study. All treatments that the Investigator considers necessary for a participant's welfare may be administered at the discretion of the Investigator in keeping with the community standards of medical care.

Medications or vaccinations specifically prohibited in the exclusion criteria are not allowed during the ongoing study. If there is a clinical indication for any medication or vaccination specifically prohibited during the study, discontinuation from study therapy or vaccination may be required. The final decision on any supportive therapy or vaccination rests with the investigator and/or the participant's primary physician. However, the decision to continue the participant on study treatment requires the mutual agreement of the investigator, the Sponsor and the participant.

There are no prohibited therapies during the Post-Treatment Follow-up Phase.

5.5.3 Rescue Medications & Supportive Care(12.2.8)

Participants should receive appropriate supportive care measures as deemed necessary by the treating investigator. Suggested supportive care measures for the management of AEs with potential immunologic etiology are outlined along with the dose modification guidelines in Section 5.2.2, [Table 3]. Where appropriate, these guidelines include the use of oral or IV treatment with corticosteroids, as well as additional anti-inflammatory agents if symptoms do not improve with administration of corticosteroids. Note that several courses of steroid tapering may be necessary as symptoms may worsen when the steroid dose is decreased. For each disorder, attempts should be made to rule out other causes such as metastatic disease or bacterial or viral infection, which might require additional supportive care. The treatment guidelines are intended to be applied when the Investigator determines the events to be related to pembrolizumab.

Note: If after the evaluation of the event, it is determined not to be related to pembrolizumab, the Investigator does not need to follow the treatment guidance. Refer to [Table 3] in Section 5.2.2 for guidelines regarding dose modification and supportive care.

It may be necessary to perform conditional procedures such as bronchoscopy, endoscopy, or skin photography as part of evaluation of the event.

5.6 Participant Withdrawal/Discontinuation Criteria(13)

Participants may discontinue study treatment at any time for any reason or be dropped from the study treatment at the discretion of the investigator should any untoward effect occur. In addition, a participant may be discontinued from study treatment by the investigator or the Sponsor if study treatment is inappropriate, the trial plan is violated, or for administrative and/or other safety reasons. Specific details regarding procedures to be performed at study treatment discontinuation are provided in Section 7.1.4 – Other Procedures.

A participant must be discontinued from study treatment but continue to be monitored in the study for any of the following reasons:

- The participant or participant's legally acceptable representative requests to discontinue study treatment

- Radiographic disease progression **documented by the investigator per RECIST 1.1 and when clinically appropriate, confirmed by the site per iRECIST** outlined in Section 7.1.2.6

  Note: An exception to continue study treatment beyond confirmed PD per iRECIST (iCPD) may be considered following consultation with the Sponsor

- Clinical progression without radiographic disease progression defined by elevated CA-125 (based on GCIG criteria) in conjunction with any of the following criteria for malignant bowel obstruction:

  - Any of the following: new or worsening abdominal pain, nausea, or vomiting
  - Abdominal distension, constipation, and/or diarrhea
  - No evidence of metabolic or electrolyte abnormalities leading to impaired intestinal motility

- Any progression or recurrence of any malignancy, or any occurrence of another malignancy that requires active treatment
- Unacceptable adverse experiences as described in Section 5.2.2.
- The participant has a medical condition or personal circumstance which, in the opinion of the investigator and/or sponsor, placed the participant at unnecessary risk from continued administration of study treatment.
- The participant interrupts pembrolizumab administration for more than 12 consecutive weeks for an AE/toxicity or for more than 3 consecutive weeks for administrative reasons without Sponsor consultation.
- The participant interrupts olaaprib for more than 4 consecutive weeks (>28 cnsecutive days) without Sponsor consultation.
- The participant has a confirmed positive serum pregnancy test
- Noncompliance with study treatment or procedure requirements
- Recurrent Grade 2 pneumonitis
- The participant is lost to follow-up
- Completion of 35 treatments (approximately 2 years) with pembrolizumab or completion of approximately 2 years of treatment with Olaparib or Participants completed maximum duration of treatment with bevacizumab according to approved for the second-line treatment of EOC, as per the local SOC and approved product label.

Note: The number of treatments is calculated starting with the first dose of pembrolizumab. And after 35 doses treatments, pembrolizumab should be discontinued.

Note: Only participants with no evidence of disease will stop treatment with olaparib following 2 years of treatment. The total duration of treatment is calculated starting with the first dose of olaparib.

5.7 Participant Replacement Strategy

Not available

5.8 Clinical Criteria for Early Trial Termination

Early trial termination will be the result of the criteria specified below:

1. Quality or quantity of data recording is inaccurate or incomplete
2. Poor adherence to protocol and regulatory requirements
3. Incidence or severity of adverse drug reaction in this or other studies indicates a potential health hazard to participants
4. Plans to modify or discontinue the development of the study drug

In the event of Merck decision to no longer supply study drug, ample notification will be provided so that appropriate adjustments to participant treatment can be made.

6.0 TRIAL FLOW CHART

6.1 Study Flow Chart

**Trial Period:**	**Screening Phase**	**Treatment Cycles**								**End of Treatment**	**Post-Treatment**		
						To be repeated beyond 8 cycles							
Treatment Cycle/Title:	Screening (Visit 1)	1	2	3	4	5	6	7	8	Discon	Safety Follow-up	Follow Up Visits	Survival Follow-Up
Scheduling Window (Days):	-28 to -1	-1	± 3	± 3	± 3	± 3	± 3	± 3	± 3	At time of Discon	30 days post discon + 7	Every 12 weeks post discon (± 7)	Every 12 weeks (± 7)
**Administrative Procedures**													
Informed Consent	X												
Inclusion/Exclusion Criteria	X												
Demographics and Medical History	X												
Prior and Concomitant Medication Review	X												
Trial Treatment Administration	X												
Post-study anticancer therapy status										X	X	X	X
Survival Status		X	X	X	X	X	X	X	X	X	X	X	X
**Clinical Procedures/Assessments**													
Review Adverse Events		X	X	X	X	X	X	X	X	X	X		
Full Physical Examination	X												
Directed Physical Examination		X	X	X	X	X	X	X	X	X	X	X	X
Vital Signs and Weight	X	X	X	X	X	X	X	X	X	X	X		
ECOG Performance Status	X	X	X	X	X	X	X	X	X	X	X		
ECG	X												
Bevacizumab		X	X	X	X	X	X	X	X				
Pembrolizumab			X	X	X	X	X	X	X				
Olaparib		X	X	X	X	X	X	X	X				
**Laboratory Procedures/Assessments: analysis performed by LOCAL laboratory**													

**Trial Period:**	**Screening Phase**	**Treatment Cycles**								**End of Treatment**	**Post-Treatment**		
Treatment Cycle/Title:	Screening (Visit 1)	1	2	3	4	To be repeated beyond 8 cycles<br>5	6	7	8	Discon	Safety Follow-up	Follow Up Visits	Survival Follow-Up
Scheduling Window (Days):	-28 to -1	-1	± 3	± 3	± 3	± 3	± 3	± 3	± 3	At time of Discon	30 days post discon + 7	Every 12 weeks post discon (± 7)	Every 12 weeks (± 7)
Pregnancy Test – Urine or Serum β-HCG	X									X			
PT/INR and aPTT	X												
CBC with Differential[b]	X	X[a]	X	X	X	X	X	X	X	X			
Comprehensive Serum Chemistry Panel[c]	X	X[a]	X	X	X	X	X	X	X	X			
Urinalysis	X	X[a]	X	X	X	X	X	X	X	X			
T3, FT4 and TSH[d]	X				X			X[a]		X			
CA-125 [e]	X				X			X					
HIV/HBV/HCV[f]	X												
**Efficacy Measurements**													
Tumor Imaging[g]	X				X			X				X	X
**Tumor Biopsies/Archival Tissue Collection/Correlative Studies Blood**													
Archival Tissue Collection	X												
Correlative Studies Blood Collection	X[h]				X[h]			X[h]		X[h]			
Tumor biopsy	X[h]		X[h]							X[h]			

Trial Period:	Screening Phase	Treatment Cycles								End of Treatment	Post-Treatment		
						To be repeated beyond 8 cycles							
Treatment Cycle/Title:	Screening (Visit 1)	1	2	3	4	5	6	7	8	Discon	Safety Follow-up	Follow Up Visits	Survival Follow-Up
Scheduling Window (Days):	-28 to -1	-1	± 3	± 3	± 3	± 3	± 3	± 3	± 3	At time of Discon	30 days post discon + 7	Every 12 weeks post discon (± 7)	Every 12 weeks (± 7)

a. If the screening test was performed within 7days prior to C1D1, additional test is not required.

b. Perform prior to each cycle as below test:
 - WBC(total) / Hemoglobin / Platelets / ANC(Absolute Neutrophil Count)

c. Perform prior to each cycle as below test:
 - Calcium / Phosphorus / Glucose / Uric acid / BUN / Creatinine / Cholesterol / Total protein / Albumin / Total bilirubin / Alkaline phosphate / AST(SGOT) / ALT(SGPT) / LDH / Sodium / Potassium / Chloride / CO2(or bicarbonate)

d. If TSH result is not within normal range, perform the T3/Free T4 test additionally.

e. After every 3 cycles of treatment, perform the test together tumor imaging.

f. Perform the test as below:
 - HIV / HBsAg / Anti-HCV

g. For participants who DC for reasons other than radiographic PD, imaging continues until radiographically documented PD by the investigator (and, when clinically appropriate, confirmed by iRECIST), initiation of a new anti-cancer therapy, withdrawal of consent, becoming lost to follow-up, pregnancy, or death, whichever occurs first. Follow-Up visits may be scheduled to coincide with Follow-Up imaging.

h. If correlative Studies Blood Collection or biopsy is feasible,

i. Refer to section 7.1.2.6.1

7.0 TRIAL PROCEDURES(17)

7.1 Trial Procedures

The Trial Flow Chart - Section 6.0 summarizes the trial procedures to be performed at each visit. Individual trial procedures are described in detail below. It may be necessary to perform these procedures at unscheduled time points if deemed clinically necessary by the investigator.

Furthermore, additional evaluations/testing may be deemed necessary by the Sponsor and/or Merck for reasons related to participant safety. In some cases, such evaluation/testing may be potentially sensitive in nature (e.g., HIV, Hepatitis C, etc.), and thus local regulations may require that additional informed consent be obtained from the participant. In these cases, such evaluations/testing will be performed in accordance with those regulations.

7.1.1 Administrative Procedures

7.1.1.1 Informed Consent

The Investigator must obtain documented consent from each potential participant prior to participating in a clinical trial.

7.1.1.1.1 General Informed Consent

Consent must be documented by the participant's dated signature or by the participant's legally acceptable representative's dated signature on a consent form along with the dated signature of the person conducting the consent discussion.

A copy of the signed and dated consent form should be given to the participant before participation in the trial.

The initial informed consent form, any subsequent revised written informed consent form and any written information provided to the participant must receive the IRB/ERC's approval/favorable opinion in advance of use. The participant or his/her legally acceptable representative should be informed in a timely manner if new information becomes available that may be relevant to the participant's willingness to continue participation in the trial. The communication of this information will be provided and documented via a revised consent form or addendum to the original consent form that captures the participant's dated signature or by the participant's legally acceptable representative's dated signature.

Specifics about a trial and the trial population will be added to the consent form template at the protocol level.

The informed consent will adhere to IRB/ERC requirements, applicable laws and regulations and Sponsor requirements.

7.1.1.2 Inclusion/Exclusion Criteria

All inclusion and exclusion criteria will be reviewed by the investigator or qualified designee to ensure that the participant qualifies for the trial.

7.1.1.3 Medical History

A medical history will be obtained by the investigator or qualified designee. Medical history will include all active conditions, and any condition diagnosed within the prior 10 years that are considered to be clinically significant by the Investigator. Details regarding the disease for which the participant has enrolled in this study will be recorded separately and not listed as medical history.

7.1.1.4 Prior and Concomitant Medications Review

7.1.1.4.1 Prior Medications

The investigator or qualified designee will review prior medication use, including any protocol-specified washout requirement, and record prior medication taken by the participant within 28 days before starting the trial. Treatment for the disease for which the participant has enrolled in this study will be recorded separately and not listed as a prior medication.

7.1.1.4.2 Concomitant Medications

The investigator or qualified designee will record medication, if any, taken by the participant during the trial. All medications related to reportable SAEs and ECIs should be recorded as defined in Section 7.2.

7.1.1.5 Disease Details and Treatments

7.1.1.5.1 Disease Details

The investigator or qualified designee will obtain prior and current details regarding disease status.

7.1.1.5.2 Prior Treatment Details

The investigator or qualified designee will review all prior cancer treatments including systemic treatments, radiation and surgeries.

7.1.1.5.3 Subsequent Anti-Cancer Therapy Status

The investigator or qualified designee will review all new anti-neoplastic therapy initiated after the last dose of trial treatment. If a participant initiates a new anti-cancer therapy within 30 days after the last dose of trial treatment, the 30 day Safety Follow-up visit must occur before the first dose of the new therapy. Once new anti-cancer therapy has been initiated the participant will move into survival follow-up.

7.1.1.6 Assignment of Screening Number

All consented participants will be given a unique screening number that will be used to identify the participant for all procedures that occur prior to enrollment. Each participant will be assigned only one screening number. Screening numbers must not be re-used for different participants.
Any participant who is screened multiple times will retain the original screening number assigned at the initial screening visit.
Specific details on the screening visit requirements (screening/rescreening) are provided in Section 7.1.1.1.1

7.1.1.7 Assignment of Randomization Number

All eligible participants will be randomly allocated and will receive a treatment/randomization number. The treatment/randomization number identifies the participant for all procedures occurring after treatment allocation/randomization. Once a treatment/randomization number is assigned to a participant, it can never be re-assigned to another participant.
A single participant cannot be assigned more than 1 treatment/randomization number.

7.1.2 Clinical Procedures/Assessments

7.1.2.1 Adverse Event (AE) Monitoring

The investigator or qualified designee will assess each participant to evaluate for potential new or worsening AEs as specified in the Trial Flow Chart and more frequently if clinically indicated. Adverse experiences will be graded and recorded throughout the study and during the follow-up period according to NCI CTCAE Version 5.0 (see Appendix 2). Toxicities will be characterized in terms regarding seriousness, causality, toxicity grading, and action taken with regard to trial treatment.

Please refer to section 7.2 for detailed information regarding the assessment and recording of AEs.

7.1.2.2 Full Physical Exam

The investigator or qualified designee will perform a complete physical exam during the screening period. Clinically significant abnormal findings should be recorded as medical history. A full physical exam should be performed during screening,

7.1.2.3 Directed Physical Exam

For cycles that do not require a full physical exam per the Trial Flow Chart, the investigator or qualified designee will perform a directed physical exam as clinically indicated prior to trial treatment administration.

7.1.2.4 Vital Signs

The investigator or qualified designee will take vital signs at screening, prior to the administration of each dose of trial treatment and at treatment discontinuation as specified in the Trial Flow Chart (Section 6.0). Vital signs should include temperature, pulse, respiratory rate, weight and blood pressure. Height will be measured at screening only.

7.1.2.5 Eastern Cooperative Oncology Group (ECOG) Performance Scale

The investigator or qualified designee will assess ECOG status (see Appendix 1) at screening, prior to the administration of each dose of trial treatment and discontinuation of trial treatment as specified in the Trial Flow Chart.

7.1.2.6 Tumor Imaging and Assessment of Disease

Tumor imaging is strongly preferred to be acquired by computed tomography (CT). For the abdomen and pelvis, contrast-enhanced magnetic resonance imaging (MRI) may be used when CT with iodinated contrast is contraindicated, or when local practice mandates it. MRI is the strongly preferred modality for imaging the brain. The same imaging technique regarding modality, ideally the same scanner, and the use of contrast should be used in a participant throughout the study to optimize the reproducibility of the assessment of existing and new tumor burden and improve the accuracy of the assessment of response or progression based on imaging.

Participant eligibility will be determined using local assessment (Investigator assessment) based on RECIST 1.1. In addition, images (including via other modalities) that are obtained at an unscheduled time point to determine disease progression, as well as imaging obtained for other reasons, but which demonstrate radiologic progression, should also be used to determine progression.

When the Investigator identifies radiographic progression per RECIST 1.1, efforts should be made to verify radiologic PD. Treatment should continue until PD has been verified. Regardless of whether PD is verified, if the Investigator considers the participant has progressed, but elects to implement iRECIST, the Investigator will assess for confirmation of progression by iRECIST at subsequent time points.

Note: For the purpose of assessing tumor imaging, the term “investigator” refers to the local investigator at the site and/or the radiological reviewer located at the site or at an offsite facility.

7.1.2.6.1 Initial Tumor Imaging

Initial tumor imaging at Screening must be performed within 28 days prior to the date of allocation. The site study team must review screening images to confirm the participant has measurable disease per RECIST 1.1.

Tumor imaging performed as part of routine clinical management is acceptable for use as screening tumor imaging if they are of diagnostic quality and performed within 28 days prior to the date of allocation.

Brain imaging, if performed to document the stability of existing metastases, should be by MRI if possible. If MRI is medically contraindicated, CT with contrast is an acceptable alternative.

7.1.2.6.2 Tumor Imaging During the Study

The first on-study imaging assessment should be performed after 3cycles (±7 days) from the date of allocation. Subsequent tumor imaging should be performed every 3cycles (±7 days) or more frequently if clinically indicated. Imaging timing should follow calendar days and should not be adjusted for delays in cycle starts. From the date of allocation, Disease status by tumor imaging is performed every 12weeks(± 7 days) from the 2 years to 3 years. After approximately 3 years, participants who remain on study and have no evidence of disease will have imaging performed every 24weeks (168 days ± 7 days). Imaging should continue to be performed until disease progression is identified by the Investigator.

Objective response should be confirmed by a repeat imaging assessment. Tumor imaging to confirm PR or CR should be performed at least 4 weeks after the first indication of a response is observed. Participants will then return to regular scheduled imaging every 3 cycles, starting with the next scheduled imaging time point. Participants who receive additional imaging for confirmation do not need to undergo the next scheduled tumor imaging if it is less than 4 weeks later; tumor imaging may resume at the subsequent scheduled imaging time point.

Per iRECIST (Section 9.2.1.6), disease progression should be confirmed by the site 4 to 8 weeks after first radiologic evidence of PD in clinically stable participants. Participants who have unconfirmed disease progression may continue on treatment at the discretion of the Investigator until progression is confirmed by the site provided they have met the conditions detailed in Section 9.2.1.6. Participants who receive confirmatory imaging do not need to undergo the next scheduled tumor imaging if it is less than 4 weeks later; tumor imaging may resume at the subsequent scheduled imaging time point, if clinically stable. Participants who have confirmed disease progression by iRECIST, as assessed by the site, will discontinue study treatment. Exceptions are detailed in Section 9.2.1.6.

7.1.2.6.3 End of Treatment and Follow-up Tumor Imaging

In participants who discontinue study treatment, tumor imaging should be performed at the time of treatment discontinuation (±4 week window). If previous imaging was obtained within 4 weeks prior to the date of discontinuation, then imaging at treatment discontinuation is not mandatory. In participants who discontinue study treatment due to documented disease progression and the Investigator elects not to implement iRECIST, this is the final required tumor imaging.

In participants who discontinue study treatment without documented disease progression, every effort should be made to continue monitoring their disease status by tumor imaging using the same imaging schedule used while on treatment (from the date of allocation, every 9 weeks in Year 2, every 12 weeks in year 3 and every 24 weeks thereafter) to monitor disease status until the start of a new anticancer treatment, disease progression, pregnancy, death, withdrawal of consent, or the end of the study, whichever occurs first.

Imaging Visit	Description
Baseline	Perform imaging within 28 days prior to allocation<br><br>To confirm CR or PR from previous platinum-based chemotherapy, imaging must be performed.
Treatment period	Imaging performed every 3 cycles (± 7days) from the date of allocation to 2 years.
Imaging For Eligibility to Continue Olaparib/ Olaparib Placebo at 2 years	Imaging should be performed within 4 weeks prior to reaching 2 years of maintenance with olaparib/olaparib placebo:<br><br>If at this scan, the participant has CR or NED, this is the final tumor imaging and the participant must discontinue olaparib/olaparib placebo.<br><br>If at this scan the participant does not have a CR and is not NED, the participant may continue olaparib/olaparib placebo until CR or NED by RECIST 1.1.
Post-treatment Follow-up imaging	Participants who discontinue study treatment without documented disease progression should continue monitoring disease status by tumor imaging Q9W (63 days ± 7days) until 2 years from the date of allocation and Q12W thereafter. After approximately 3 years, participants who remain on study and have no evidence of disease will have imaging performed every 24 weeks (168 days ± 7 days).
End of Treatment	Imaging performed at the time of treatment discontinuation (±4 weeks). If previous imaging was obtained within 4 weeks before the date of discontinuation, then imaging at treatment discontinuation is not mandatory. For participants who discontinue study treatment due to documented PD, this is the final required tumor imaging if the investigator elects not to implement iRECIST.
Abbreviations: CR = complete response; iRECIST = modified Response Evaluation Criteria in Solid Tumors 1.1 for immune-based therapeutics; PD = progressive disease; PR = partial response	

7.1.2.6.4 RECIST 1.1 Assessment of Disease

RECIST 1.1 will be used as the primary measure for assessment of tumor response, date of disease progression, and as a basis for all protocol guidelines related to disease status (eg, discontinuation of study treatment). Although RECIST 1.1 references a maximum of 5 target lesions in total and 2 per organ, the Sponsor allows a maximum of 10 target lesions in total and 5 per organ, if clinically relevant to enable a broader sampling of tumor burden. The first

half of the flowchart in [Figure 3] illustrates the imaging flow involving verification of PD for clinically stable participants.

7.1.2.6.5 iRECIST Assessment of Disease

iRECIST is based on RECIST 1.1, but adapted to account for the unique tumor response seen with immunotherapeutic drugs. When clinically stable, participants should not be discontinued until progression is confirmed by the Investigator, working with local radiology, according to the rules below. This allowance to continue treatment despite initial radiologic PD takes into account the observation that some participants can have a transient tumor flare in the first few months after the start of immunotherapy, and then experience subsequent disease response.

A description of the adaptations and iRECIST process is provided in Appendix 4, with additional detail in the iRECIST publication [Seymour et al, 2017]. iRECIST will be used by the Investigator to assess tumor response and progression, and make treatment decisions.

Table 9 Imaging and Treatment after First Radiologic Evidence of Progressive Disease

	**Clinically Stable**		**Clinically Unstable**	
	**Imaging**	**Treatment**	**Imaging**	**Treatment**
First radiologic evidence of PD by RECIST 1.1	Repeat imaging at 4 to 8 weeks to confirm PD.	May continue study treatment at the Investigator's discretion while awaiting confirmatory tumor imaging by site by iRECIST.	Repeat imaging at 4 to 8 weeks to confirm PD per Investigator's discretion only.	Discontinue treatment
Repeat tumor imaging confirms PD (iCPD) by iRECIST per Investigator assessment	No additional imaging required.	Discontinue treatment (exception is possible upon consultation with Sponsor).	No additional imaging required.	Not applicable
Repeat tumor imaging shows iUPD by iRECIST per Investigator assessment	Repeat imaging at 4 to 8 weeks to confirm PD. May occur at next regularly scheduled imaging visit.	Continue study treatment at the Investigator's discretion.	Repeat imaging at 4 to 8 weeks to confirm PD per Investigator's discretion only.	Discontinue treatment
Repeat tumor imaging shows iSD, iPR, or iCR by iRECIST per Investigator assessment.	Continue regularly scheduled imaging assessments.	Continue study treatment at the Investigator's discretion.	Continue regularly scheduled imaging assessments.	May restart study treatment if condition has improved and/or clinically stable per Investigator's discretion. Next tumor image should occur according to the regular imaging schedule.

iCPD = iRECIST confirmed progressive disease; iCR = iRECIST complete response; iRECIST = modified Response Evaluation Criteria in Solid Tumors 1.1 for immune-based therapeutics; iSD = iRECIST stable disease; iUPD = iRECIST unconfirmed progressive disease; PD = progressive disease; RECIST 1.1 = Response Evaluation Criteria in Solid Tumors 1.1..

Figure 3: Imaging and Treatment for Clinically Stable Participants after First Radiologic Evidence of PD Assessed by the Investigator

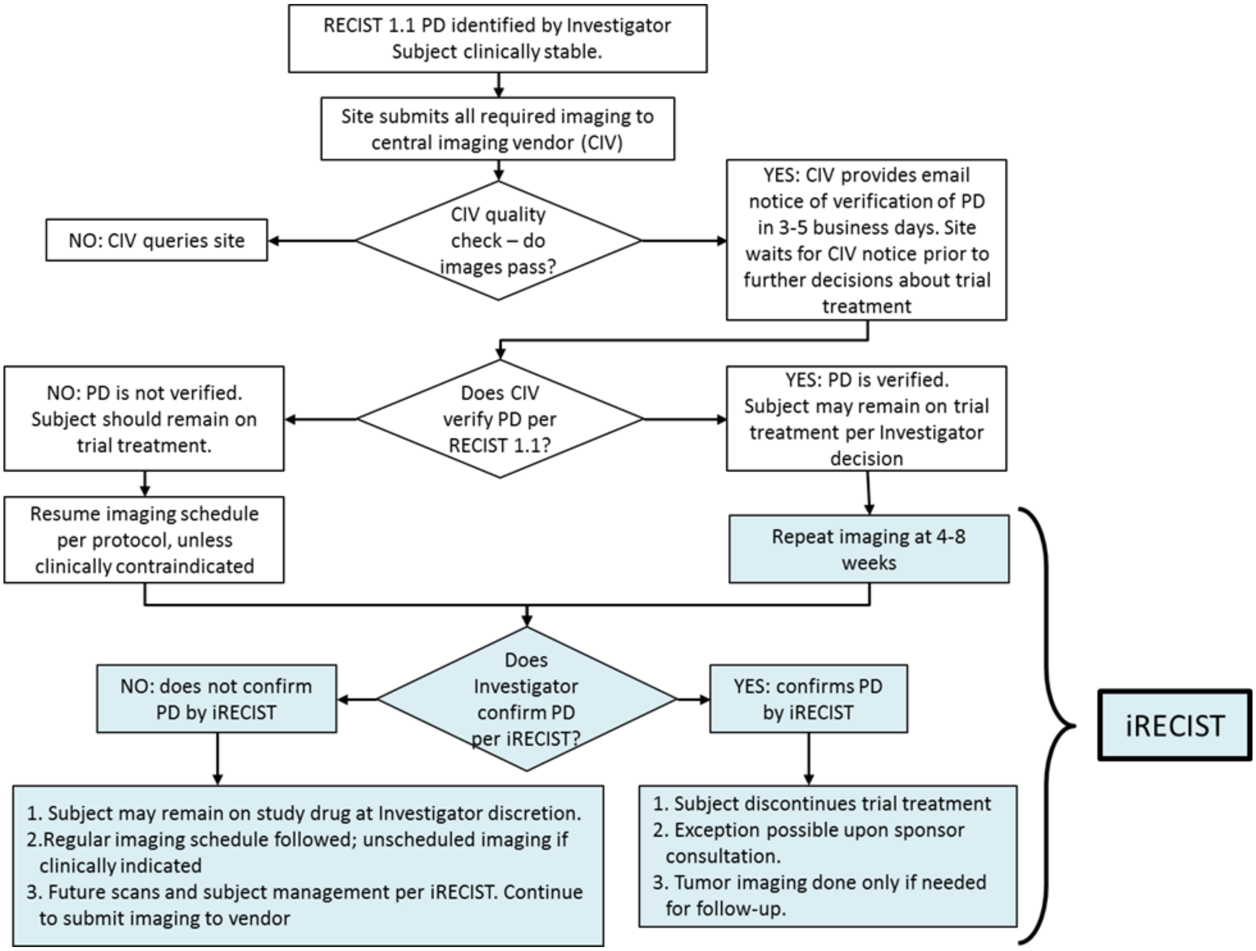

7.1.2.7 Tumor Tissue Collection and Correlative Studies Blood Sampling

Participant in this study will be dependent upon participants supplying tumor tissue and blood for biomarker analysis. An archival tissue and/or pre, on, post-treatment core needle or excision biopsy is preferred. Submission of either FFPE tumor blocks or unstained slides for archival tissue and pre-chemotherapy sample is acceptable. Pre-treatment and/or on-treatment biopsy should be obtained prior to administration of study drug and 3 weeks after olaparib.

- Collection of archival samples (FFPE or fresh-frozen)
- Pre-chemotherapy radiological guided biopsy (3 cores) if lesions are deemed safe and accessible by the investigator
- Pre-treatment & On-treatment radiological guided biopsy (3 cores) if lesions are deemed safe and accessible by the investigator

- Blood will be collected for ctDNA, PBMC, and plasma anlaysis. ctDNA, PBMC, and plasma

Participants must sign the main study ICF prior to submitting existing tissue samples and/or undergoing a new biopsy.

Archival samples, pre-treatment/ on-treatment sample, and post-treatment sample will be submitted. Blood samples every 3 weeks during treatment period will be collected.

Comprehensive genomic profiling & immune biomarker exploration (exome sequencing, RNA sequencing, and immunohistochemistry) will be performed for all samples to find the predictive biomarkers for combination therapy of olaparib, bevacizumab, and pembrolizumab.

- Responder for 3 combination therapy

BRCA reversion and HRD phenotype changes will be checked through serial samples. (somatic HR mutations, germline HR mutations, altered gene expression, functional HR deficiency, genomic scars, RAD51 foci formation).

BRCA reversion mutations will be checked through circulating tumor DNA serial samples

- Immune dynamic change

RNA sequencing will be performed to identify immune dynamic changes between at pre-treatment and at progression.

- Analysis of immune marker using FACS

FACS will be performed to examine the phenotypic characteristics of CD4 and CD8 T cells in terms of various immune check points (PD-1, CTLA4, TIM3, LAG3) and memory markers (CD45RO+CCR7+) in fresh tissue and PBMC.

tumor infiltrating immune cells including Treg cells and MDSCs will be evaluated on pre-treatment & at progression.

- PD-L1 testing and multiplex IHC

PD-L1 expression level will be checked through serial samples. TPS and CPS will be calculated.

Using established platform of Vectra, multispectral imaging will be performed to identify tumor microenvironment compresensively.

- Blood samples

From C1D1 blood samples, ctDNA, PBMCs and plasma will be isolated and stored for exploratory biomarker analysis. Serial blood samples will be collected every 3 weeks during treatment period for ctDNA.

7.1.3 Laboratory Procedures/Assessments

Details regarding specific laboratory procedures/assessments to be performed in this trial are provided below.

Laboratory tests for hematology, chemistry, urinalysis, and others are specified in Table 10.

Table 10 Laboratory Tests

Hematology	Chemistry	Urinalysis	Other
WBC (total and differential)	Albumin	Blood	Serum β-human chorionic gonadotropin†
Hemoglobin	Alkaline phosphatase	Glucose	(β-hCG)[a]
Platelet count	Alanine aminotransferase (ALT)	Protein	PT (INR)
Absolute Neutrophil Count	Aspartate aminotransferase (AST)	Specific gravity	aPTT
	Lactate dehydrogenase (LDH)	Microscopic exam *(If abnormal)*	Total thriiodothyronine (T3)[c]
	Carbon Dioxide [b]*(CO_2 or biocarbonate)*	results are noted	Free tyroxine (T4) [c]
	Uric Acid	Urine pregnancy test [a]	Thyroid stimulating hormone (TSH)
	Calcium		CA-125
	Cholesterol		HIV
	Chloride		HBsAg
	Glucose		Anti-HCV
	Phosphorus		
	Potassium		Blood for correlative studies
	Sodium		
	Magnesium		
	Total Bilirubin		
	Direct Bilirubin *(If total bilirubin is elevated above the upper limit of normal)*		
	Total protein		
	Blood Urea Nitrogen		

a. Perform on women of childbearing potential only. If urine pregnancy results cannot be confirmed as negative, a serum pregnancy test will be required.
b. If considered standard of care in your region.
c. If TSH result is not within normal range, perform the T3/Free T4 test additionally.

Laboratory tests for screening or entry into the Second Course Phase should be performed within 10 days prior to the first dose of treatment. After Cycle 1, pre-dose laboratory procedures can be conducted up to 72 hours prior to dosing. Results must be reviewed by the investigator or qualified designee and found to be acceptable prior to each dose of trial treatment.

7.1.4 Other Procedures

7.1.4.1 Withdrawal/Discontinuation

When a participant discontinues/withdraws prior to trial completion, all applicable activities scheduled for the final trial visit should be performed at the time of discontinuation. Any adverse events which are present at the time of discontinuation/withdrawal should be followed in accordance with the safety requirements outlined in Section 7.2 - Assessing and Recording Adverse Events. Participants who a) attain a CR or b) complete 24 months of treatment with pembrolizumab may discontinue treatment with the option of restarting treatment if they meet the criteria specified in Section 5.2.3. After discontinuing treatment following assessment of CR, these participants should return to the site for a Safety Follow-up Visit (described in Section 7.1.5.3.1) and then proceed to the Follow-Up Period of the study (described in Section 7.1.5.3.2).

7.1.4.2 Blinding/Unblinding

Not available

7.1.5 Visit Requirements

Visit requirements are outlined in Section 6.0 - Trial Flow Chart. Specific procedure-related details are provided above in Section 7.1 - Trial Procedures.

7.1.5.1 Screening

7.1.5.1.1 Screening Period

Within 28 days prior to allocation, potential participants will be evaluated to determine that they fulfill the entry requirements as set forth in Section 5.
Written consent must be obtained prior to performing any protocol-specific procedure.
Results of a test performed prior to the participant signing consent as part of routine clinical management are acceptable in lieu of a screening test if performed within the specified time frame. Screening procedures are to be completed within 28-day Screening Period, except for the following:

- Laboratory tests are to be performed within 7 days prior to the initiating study treatment.

An exception is HIV and hepatitis testing which may be done up to 28 days prior to the first dose of study treatment if required by the local health authority.

o Evaluation of ECOG is to be performed within 7 days prior to initiating within 7 days of allocation.
o For WOCBP, a urine pregnancy test will be performed within 72 hours prior to initiating allocation. If urine pregnancy results cannot be confirmed as negative, a serum pregnancy test will be required (performed by the local study site laboratory).
o Newly obtained tissue may be obtained optionally at any time prior to the administration of investigational product.

Participants may be rescreened after initially failing to meet the inclusion/exclusion criteria. Results from assessments during the initial Screening Period are acceptable in lieu of a repeat screening test if performed within the specified time frame and the corresponding inclusion/exclusion criteria is met. Participants who are rescreened will retain their original screening number.

7.1.5.2 Treatment Period

Visit requirements are outline in the study flow chart. Specific procedure-related details are provided in Section

7.1.5.3 Post-Treatment Visits

7.1.5.3.1 Safety Follow-Up Visit

The mandatory Safety Follow-Up Visit should be conducted approximately 30 days after the last dose of study treatment or before the initiation of a new anti-cancer treatment, whichever comes first. All AEs that occur prior to the Safety Follow-Up Visit should be recorded. Participants with an AE of Grade > 1 will be followed until the resolution of the AE to Grade 0-1 or until the beginning of a new anti-cancer therapy, whichever occurs first. SAEs that occur within 90 days of the end of treatment or before initiation of a new anti-cancer treatment should also be followed and recorded.

7.1.5.3.2 Follow-up Visits

Participants who discontinue study treatment for a reason other than disease progression will move into the Follow-Up Phase and should be assessed imaging Q9W(63 days ± 7days) until 2 years from the date of allocation and Q12W thereafter. After approximately 3 years, participants who remain on
study and have no evidence of disease will have imaging performed every 24weeks (168 days ± 7 days).Every effort should be made to collect information regarding disease status until the start of new anti-cancer therapy, disease progression, death, end of the study or if the participant begins retreatment with pembrolizumab as detailed in Section 5.2.3. Information regarding post-study anti-cancer treatment will be collected if new treatment is initiated.

7.1.5.3.3 Survival Follow-up

Participants who experience confirmed disease progression or start a new anticancer therapy, will move into the Survival Follow-Up Phase and should be contacted by telephone every 12 weeks to assess for survival status until death, withdrawal of consent, or the end of the trial, whichever occurs first.

7.2 Adverse Events (AEs), Serous Adverse Events (SAEs), and Other reportable Safety Events

The definitions of an AE or SAE, as well as the method of recording, evaluating, and assessing causality of AE and SAE and the procedures for completing and transmitting AE, SAE, and other reportable safety event reports can be found in Appendix 5.

Adverse events, SAEs, and other reportable safety events will be reported by the participant (or, when appropriate, by a caregiver, surrogate, or the participant's legally authorized representative).

The investigator and any designees are responsible for detecting, documenting, and reporting events that meet the definition of an AE or SAE as well as other reportable safety events. Investigators remain responsible for following up AEs, SAEs, and other reportable safety events for outcome.

The investigator, who is a qualified physician, will assess events that meet the definition of an AE or SAE as well as other reportable safety events with respect to seriousness, intensity/toxicity and causality.

7.2.1 Time Period and Frequency for Collecting AE, SAE, and Other Reportable Safety Event Information

All AEs, SAEs, and other reportable safety events that occur after the consent form is signed but before intervention allocation/randomization must be reported by the investigator if the participant is receiving placebo run-in or other run-in treatment, if the event cause the participant to be excluded from the study, or is the result of a protocol-specified intervention, including but not limited to washout or discontinuation of usual therapy, diet, or a procedure.

- All AEs from the time of intervention allocation/randomization through 30 days following cessation of study intervention must be reported by the investigator.
- All AEs meeting serious criteria, from the time of intervention allocation/randomization through 90 days following cessation of study intervention or 30 days following cessation of study intervention if the participant initiates new anticancer therapy, whichever is earlier, must be reported by the investigator.

- All pregnancies and exposure during breastfeeding, from the time of intervention allocation/randomization through 120 days following cessation of study intervention, or 30 days following cessation of study intervention if the participant initiates new anticancer therapy must be reported by the investigator.
- Additionally, any SAE brought to the attention of an investigator at any time outside of the time period specified above must be reported immediately to Merck if the event is considered drug-related.

Investigators are not obligated to actively seek AEs or SAEs or other reportable safety events in former study participants. However, if the investigator learns of any SAE, including a death, at any time after a participant has been discharged from the study, and he/she considers the event to be reasonably related to the study intervention or study participation, the investigator must promptly notify Merck.

All initial and follow-up AEs, SAEs, and other reportable safety events will be recorded and reported to Merck within the time frames as indicated in Table 11.

Table 11 Reporting Time Periods and Time Frames for Adverse Events and Other Reportable Safety Events

**Type of Event**	**Reporting Time Period:** Consent to Randomization/ Allocation	**Reporting Time Period:** Randomization/ Allocation through Protocol-specified Follow-up Period	**Reporting Time Period:** After the Protocol-specified Follow-up Period	**Time Frame to Report Event and Follow-up Information to Merck:**
Serious Adverse Event (SAE) including Cancer and Overdose	Report if:<br>- due to protocol-specified intervention<br>- causes exclusion<br>- participant is receiving placebo run-in or other run-in treatment	Report all	Report if:<br>- drug/vaccine related.<br>(Follow ongoing to outcome)	Within 2 business days but no longer than 3 calendar days of learning of event
Pregnancy/Lactation Exposure	Report if:<br>- due to intervention<br>- causes exclusion	Report all	Previously reported – Follow to completion/termination; report outcome	Within 2 business days but no longer than 3 calendar days of learning of event
Event of Clinical Interest (require regulatory reporting)	Report if:<br>- due to intervention<br>- causes exclusion	Report<br>- potential drug-induced liver injury (DILI)	Not required	Within 2 business days but no longer than 3 calendar days

**Type of Event**	<u>**Reporting Time Period:**</u> **Consent to Randomization/ Allocation**	<u>**Reporting Time Period:**</u> **Randomization/ Allocation through Protocol-specified Follow-up Period**	<u>**Reporting Time Period:**</u> **After the Protocol-specified Follow-up Period**	**Time Frame to Report Event and Follow-up Information to Merck:**
		- require regulatory reporting		of learning of event

7.2.2 Method of Detecting AEs, SAEs, and Other Reportable Safety Events

Care will be taken not to introduce bias when detecting AEs and/or SAEs and other reportable safety events. Open-ended and nonleading verbal questioning of the participant is the preferred method to inquire about AE occurrence.

7.2.3 Follow-up of AE, SAE, and Other Reportable Safety Event Information

After the initial AE/SAE report, the investigator is required to proactively follow each participant at subsequent visits/contacts. All AEs, SAEs, and other reportable safety events including pregnancy and exposure during breastfeeding, events of clinical interest (ECIs), cancer, and overdose will be followed until resolution, stabilization, until the event is otherwise explained, or the participant is lost to follow-up. In addition, the investigator will make every attempt to follow all nonserious AEs that occur in randomized participants for outcome. Further information on follow-up procedures is given in Appendix 5.

7.2.4 Sponsor Responsibility for Reporting Adverse Events

All Adverse Events will be reported to regulatory authorities, IRB/IECs and investigators in accordance with all applicable country specific regulatory requirements, global laws and regulations.

7.2.5 Pregnancy and Exposure During Breastfeeding

Although pregnancy and infant exposure during breastfeeding are not considered AEs, any pregnancy or infant exposure during breastfeeding in a participant (spontaneously reported to the investigator or their designee) that occurs during the study are reportable to Merck.

All reported pregnancies must be followed to the completion/termination of the pregnancy. Pregnancy outcomes of spontaneous abortion, missed abortion, benign hydatidiform mole, blighted ovum, fetal death, intrauterine death, miscarriage, and stillbirth must be reported as serious events (Important Medical Events). If the pregnancy continues to term, the outcome (health of infant) must also be reported.

7.2.6 Events of Clinical Interest (ECIs)

Selected nonserious and SAEs are also known as ECIs and must be reported to Merck.

Events of clinical interest for this study include:

1. An overdose of pembrolizumab that is not associated with clinical symptoms or abnormal laboratory results. For purposes of this study, an overdose of pembrolizumab will be defined as any dose of 1,000 mg or greater (≥5 times the indicated dose). No specific information is available on the treatment of overdose of pembrolizumab. In the event of overdose, the participant should be observed closely for signs of toxicity. Appropriate supportive treatment should be provided if clinically indicated. If an adverse event(s) is associated with ("results from") the overdose of a Merck product, the adverse event(s) is reported as a serious adverse event, even if no other seriousness criteria are met.

2. An elevated AST or ALT lab value that is greater than or equal to 3X the upper limit of normal and an elevated total bilirubin lab value that is greater than or equal to 2X the upper limit of normal and, at the same time, an alkaline phosphatase lab value that is less than 2X the upper limit of normal, as determined by way of protocol-specified laboratory testing or unscheduled laboratory testing.*

   *Note: These criteria are based upon available regulatory guidance documents. The purpose of the criteria is to specify a threshold of abnormal hepatic tests that may require an additional evaluation for an underlying etiology.

8.0 STATISTICAL ANALYSIS PLAN

8.1 Statistical Analysis Plan Summary

The sample size was calculated based on a Simon's Two-stage optimal design with assumptions concerning the estimated PFS rate in ovarian cancer.

The rate of patients with disease-free state at 6 months is expected as 50% with bevacizumab maintenance (current SOC, results from GOG 213), and HR of adding maintenance therapy of 3 combinations (PARP inhibitor, antiangiogenic therapy, and immune checkpoint inhibitor) was assumed to 0.5, which was equivalent to 70.7% of PFS rate. When applying the same expected efficacy (HR=0.5) with DUO-O study (NCT0373764), the null hypothesis for this study will be 50% of 6 month PFS rate, and alternative hypothesis of interest will be 70% of 6 month PFS rate.

Using Simon's two stage optimal design at a one-sided 5% level of significant and 80% power, totally 39 patients are required in this study. In 1st stage, 22 patients will be enrolled, then if 12

or fewer respond, the trial will be terminated. If not, the trial continues to the 2nd stage, a total of 39 patients will be studies. If the total number responding is less than or equal to 24, the alternative hypothesis is rejected.

Considering 10% follow-up loss, the sample size will be 44 patients in each dose.

8.2 Statistical Analysis Plan

- Efficacy analyses are based on the modified intent-to-treat (ITT) approach (patients should receive at least one treatment dose). The final analysis of 6-months PFS will be analyzed the proportion and number of patients who are alive and progression-free 6-months after the first dose of study therapy. Overall survival(OS) and Progression Free Survival(PFS) are will be provided the mean and median, it will be provided the mean and median to subsequent chemotherapy if the patient has been administered subsequent chemotherapy.
- Safety analyses are based on the safety population (at least one dose of study drug). Adverse events are graded according to CTCAE version 5.0. It will be estimated to be separated into hematologic AE and non-hematologic AE. Also it will be conduct to analysis for all adverse events, grade 3 or higher, adverse events that cannot be excluded from causal relationship with investigational product, and serious adverse event. It will be provided the proportion of subject that significant changes from baseline in clinical laboratory, vital sign, and physical examination by treatment.
- Interim After recruiting 22 patients, interim analysis to determine futility of the treatment will be conducted.
- Missing data will not be replaced by other data, and if tumor imaging assessment was missed, disease progression or death, if new anticancer therapy has been initiated, the data will be censored at the last assessment.
- Descriptive statistics will be used to summarize the characteristics of the study patients.
- Survival analyses are performed using Kaplan-Meier method.

9.0 LABELING, PACKAGING, STORAGE AND RETURN OF CLINICAL SUPPLIES

9.1 Investigational Product

The investigator shall take responsibility for and shall take all steps to maintain appropriate records and ensure appropriate supply, storage, handling, distribution and usage of investigational product in accordance with the protocol and any applicable laws and regulations.

Pembrolizumab and Olaparib will be provided by Merck as summarized in Table 12.

Table 12 Product Descriptions

Product Name & Potency	Dosage Form
Pembrolizumab 100 mg/ 4mL	Solution for Injection
Olaparib 100mg, 150mg	Tablet

9.2 Packaging and Labeling Information

Supplies will be labeled in accordance with regulatory requirements.

9.3 Clinical Supplies Disclosure

This trial is open-label; therefore, the participant, the trial site personnel, the Sponsor and/or designee are not blinded to treatment. Drug identity (name, strength) is included in the label text; random code/disclosure envelopes or lists are not provided.

9.4 Storage and Handling Requirements

Clinical supplies must be stored in a secure, limited-access location under the storage conditions specified on the label.

Receipt and dispensing of trial medication must be recorded by an authorized person at the trial site.

Clinical supplies may not be used for any purpose other than that stated in the protocol.

9.5 Returns and Reconciliation

The investigator is responsible for keeping accurate records of the clinical supplies received from Merck or designee, the amount dispensed to and returned by the participants and the amount remaining at the conclusion of the trial.

Upon completion or termination of the study, all unused and/or partially used investigational product will be destroyed at the site per institutional policy. It is the Investigator's responsibility to arrange for disposal of all empty containers, provided that procedures for proper disposal have been established according to applicable federal, state, local and institutional guidelines and procedures, and provided that appropriate records of disposal are kept.

10.0 ADMINISTRATIVE AND REGULATORY DETAILS

Not available

11.0 REFERENCES

Ame JC, Spenlehauer C, de Murcia G. The PARP superfamily. Bioessays. 2004 Aug;26(8):882-93.

Disis ML. Immune regulation of cancer. J Clin Oncol 2010;28(29):4531-8.

Dudley ME, Wunderlich JR, Yang JC, Sherry RM, Topalian SL, Restifo NP, et al. Adoptive cell transfer therapy following non-myeloablative but lymphodepleting chemotherapy for the treatment of patients with refractory metastatic melanoma. J Clin Oncol 2005;23(10):2346-57.

Fong PC, Boss DS, Yap TA, Tutt A, Wu P, Mergui-Roelvink M, et al. Inhibition of Poly(ADP-Ribose) polymerase in tumors from BRCA mutation carriers. N Engl J Med 2009;361(2):123-34.

George A, Kaye S, Banerjee S. Delivering widespread BRCA testing and PARP inhibition to patients with ovarian cancer. Nat Rev Clin Oncol. 2017 May;14(5):284-296.

Gonzalez-Martin, A, et al. Niraparib in Patients with Newly Diagnosed Advanced Ovarian Cancer. N Engl J Med, 2019. 381(25): p. 2391-2402.

Greenwald RJ, Freeman GJ, Sharpe AH. The B7 family revisited. Annu Rev Immunol 2005;23:515-48.

Hunder NN, Wallen H, Cao J, Hendricks DW, Reilly JZ, Rodmyre R, et al. Treatment of metastatic melanoma with autologous CD4+ T cells against NY-ESO-1. N Engl J Med 2008;358(25):2698-703.

Konstantinopoulos, P.A, et al. Single-Arm Phases 1 and 2 Trial of Niraparib in Combination With Pembrolizumab in Patients With Recurrent Platinum-Resistant Ovarian Carcinoma. JAMA Oncol, 2019.

Ledermann J, Harter P, Gourley C, Friedlander M, Vergote I, Rustin G, et al. Olaparib maintenance therapy in platinum sensitive relapsed ovarian cancer. N Engl J Med. 2012 Apr 12;366(15):1382-92.

Liu JF, Barry WT, Birrer M, et al. Combination cediranib and olaparib versus olaparib alone for women with recurrent platinum-sensitive ovarian cancer: a randomised phase 2 study. The Lancet Oncology 2014;15:1207-14.

Makker, V, et al. Lenvatinib plus pembrolizumab in patients with advanced endometrial cancer: an interim analysis of a multicentre, open-label, single-arm, phase 2 trial. Lancet Oncol, 2019. 20(5): p. 711-718.

Mirza, M.R, et al. Niraparib plus bevacizumab versus niraparib alone for platinum-sensitive recurrent ovarian cancer (NSGO-AVANOVA2/ENGOT-ov24): a randomised, phase 2, superiority trial. Lancet Oncol, 2019. 20(10): p. 1409-1419.

Okazaki T, Maeda A, Nishimura H, Kurosaki T, Honjo T. PD-1 immunoreceptor inhibits B cell receptor-mediated signaling by recruiting src homology 2-domain-containing tyrosine phosphatase 2 to phosphotyrosine. Proc Natl Acad Sci U S A 2001;98(24):13866-71.

Prakash R, Zhang Y, Feng W, Jasin M. Homologous recombination and human health: the roles of BRCA1, BRCA2, and associated proteins. Cold Spring Harb Perspect Biol. 2015;7:a016600.

Pujade-Lauraine E, Ledermann JA, Selle F, Gebski V, Penson RT, Oza AM, Korach J, et al. Olaparib tablets as maintenance therapy in patients with platinum-sensitive, relapsed ovarian cancer and a BRCA1/2 mutation (SOLO2/ENGOT-Ov21): a double-blind, randomised, placebo controlled, phase 3 trial. Lancet Oncol. 2017 Sep;18(9):1274-1284.

Ray-Coquard, I, et al. Olaparib plus Bevacizumab as First-Line Maintenance in Ovarian Cancer. N Engl J Med, 2019. 381(25): p. 2416-2428.

Zhang X, Schwartz J-CD, Guo X, Bhatia S, Cao E, Chen L, et al. Structural and functional analysis of the costimulatory receptor programmed death-1. Immunity 2004;20:337-47.

Chemnitz JM, Parry RV, Nichols KE, June CH, Riley JL. SHP-1 and SHP-2 associate with immunoreceptor tyrosine-based switch motif of programmed death 1 upon primary human T cell stimulation, but only receptor ligation prevents T cell activation. J Immunol 2004;173:945-54.

Sheppard K-A, Fitz LJ, Lee JM, Benander C, George JA, Wooters J, et al. PD-1 inhibits T-cell receptor induced phosphorylation of the ZAP70/CD3zeta signalosome and downstream signaling to PKCtheta. FEBS Lett. 2004;574:37-41.

Riley JL. PD-1 signaling in primary T cells. Immunol Rev 2009;229:114-25.

Parry RV, Chemnitz JM, Frauwirth KA, Lanfranco AR, Braunstein I, Kobayashi SV, et al. CTLA-4 and PD-1 receptors inhibit T-cell activation by distinct mechanisms. Mol Cell Biol 2005;25(21):9543-53.

Francisco LM, Sage PT, Sharpe AH. The PD-1 pathway in tolerance and autoimmunity. Immunol Rev 2010;236:219-42.

Seymour L, Bogaerts J, Perrone A, Ford R, Schwartz LH, Mandrekar S, et al. iRECIST: guidelines for response criteria for use in trials testing immunotherapeutics. Lancet Oncol. 2017;18(3):e143-e152. Epub 2017 Mar 2.

12.0 APPENDICES

Appendix 1: ECOG Performance Status

Grade	Description
0	Normal activity. Fully active, able to carry on all pre-disease performance without restriction.
1	Symptoms, but ambulatory. Restricted in physically strenuous activity, but ambulatory and able to carry out work of a light or sedentary nature (e.g., light housework, office work).
2	In bed <50% of the time. Ambulatory and capable of all self-care, but unable to carry out any work activities. Up and about more than 50% of waking hours.
3	In bed >50% of the time. Capable of only limited self-care, confined to bed or chair more than 50% of waking hours.
4	100% bedridden. Completely disabled. Cannot carry on any self-care. Totally confined to bed or chair.
5	Dead.

* As published in Am. J. Clin. Oncol.: *Oken, M.M., Creech, R.H., Tormey, D.C., Horton, J., Davis, T.E., McFadden, E.T., Carbone, P.P.: Toxicity And Response Criteria Of The Eastern Cooperative Oncology Group. Am J Clin Oncol 5:649-655, 1982.* The Eastern Cooperative Oncology Group, Robert Comis M.D., Group Chair.

Appendix 2: Common Terminology Criteria for Adverse Events V5.0 (CTCAE)

The descriptions and grading scales found in the revised NCI Common Terminology Criteria for Adverse Events (CTCAE) version 5.0 will be utilized for adverse event reporting. (http://ctep.cancer.gov/reporting/ctc.html)

Appendix 3: Contraceptive Guidance and Pregnancy Testing

Woman of Childbearing Potential (WOCBP)

A woman is considered fertile following menarche and until becoming post-menopausal unless permanently sterile (see below)

Women in the following categories are not considered WOCBP:

- Premenarchal
- Premenopausal female with 1 of the following:
  - Documented hysterectomy
  - Documented bilateral salpingectomy
  - Documented bilateral oophorectomy

  Note: Documentation can come from the site personnel's review of the participant's medical records, medical examination, or medical history interview.

- Postmenopausal female
  - A postmenopausal state is defined as no menses for 12 months without an alternative medical cause.
    - A high follicle stimulating hormone (FSH) level in the postmenopausal range may be used to confirm a postmenopausal state in women not using hormonal contraception or hormonal replacement therapy (HRT). However, in the absence of 12 months of amenorrhea, confirmation with two FSH measurements in the postmenopausal range is required.
  - Females on HRT and whose menopausal status is in doubt will be required to use one of the non-hormonal highly effective contraception methods if they wish to continue their HRT during the study. Otherwise, they must discontinue HRT to allow confirmation of postmenopausal status before study enrollment.

Contraception Requirements

Female Participants:

Female participants of childbearing potential are eligible to participate if they agree to use a highly effective method of contraception consistently and correctly as described in Table 13 during the protocol-defined time frame in Section X.

Table 13 Highly Effective Contraception Methods

**Highly Effective Contraceptive Methods That Are User Dependent** [a]<br>*Failure rate of <1% per year when used consistently and correctly.*

● Combined (estrogen- and progestogen- containing) hormonal contraception [b, c]<br>○ Oral<br>○ Intravaginal<br>○ Transdermal<br>○ Injectable
● Progestogen-only hormonal contraception [b, c]<br>○ Oral<br>○ Injectable
**Highly Effective Methods That Have Low User Dependency**<br>*Failure rate of <1% per year when used consistently and correctly.*
● Progestogen- only contraceptive implant [b, c]<br>● Intrauterine hormone-releasing system (IUS) [b]<br>● Intrauterine device (IUD)<br>● Bilateral tubal occlusion
● **Vasectomized partner**<br>A vasectomized partner is a highly effective contraception method provided that the partner is the sole male sexual partner of the WOCBP and the absence of sperm has been confirmed. If not, an additional highly effective method of contraception should be used.
● **Sexual abstinence**<br>Sexual abstinence is considered a highly effective method only if defined as refraining from heterosexual intercourse during the entire period of risk associated with the study treatment. The reliability of sexual abstinence needs to be evaluated in relation to the duration of the study and the preferred and usual lifestyle of the participant.)
Notes:<br>Use should be consistent with local regulations regarding the use of contraceptive methods for participants of clinical studies.<br><br>a) Typical use failure rates are lower than perfect-use failure rates (i.e. when used consistently and correctly).<br>b) If hormonal contraception efficacy is potentially decreased due to interaction with study treatment, condoms must be used in addition to the hormonal contraception during the treatment period and for at least 120 days following the last dose of pembrolizumab and olaparib and at least following the last dose of bevacizumab.<br>c) If locally required, in accordance with Clinical Trial Facilitation Group (CTFG) guidelines, acceptable hormonal contraceptives are limited to those which inhibit ovulation.

Pregnancy Testing

WOCBP should only be included after a negative highly sensitive urine or serum pregnancy test.

Following initiation of treatment, pregnancy testing will be performed whenever an expected menstrual cycle is missed or when pregnancy is otherwise suspected; at the time points specified in the Schedule of Activities, and as required locally.

Pregnancy testing will be performed whenever an expected menstrual cycle is missed or when pregnancy is otherwise suspected.

Appendix 4: Description of the iRECIST Process for Assessment of Disease Progression

Assessment at Screening and Prior to RECIST 1.1 Progression

Until radiographic progression based on RECIST 1.1, there is no distinct iRECIST assessment.

Assessment and Decision at RECIST 1.1 Progression

In participants who show evidence of radiological PD by RECIST 1.1 the Investigator will decide whether to continue a participant on study treatment until repeat imaging is obtained (using iRECIST for participant management (see Table 5 and Figures 1 and 3). This decision by the Investigator should be based on the participant's overall clinical condition.

Clinical stability is defined as the following:

- Absence of symptoms and signs indicating clinically significant progression of disease
- No decline in ECOG performance status
- No requirements for intensified management, including increased analgesia, radiation, or other palliative care

Any participant deemed clinically unstable should be discontinued from study treatment at site-assessed first radiologic evidence of PD, and is not required to have repeat tumor imaging for confirmation of PD by iRECIST.

If the Investigator decides to continue treatment, the participant may continue to receive study treatment and the tumor assessment should be repeated 4 to 8 weeks later to confirm PD by iRECIST, per Investigator assessment.

Tumor flare may manifest as any factor causing radiographic progression per RECIST 1.1, including:

- Increase in the sum of diameters of target lesion(s) identified at baseline to ≥ 20% and ≥ 5 mm from nadir
    - Please note: the iRECIST publication uses the terminology "sum of measurements", but "sum of diameters" will be used in this protocol, consistent with the original RECIST 1.1 terminology.
- Unequivocal progression of non-target lesion(s) identified at baseline
- Development of new lesion(s)

iRECIST defines new response categories, including iUPD (unconfirmed progressive disease) and iCPD (confirmed progressive disease). For purposes of iRECIST assessment,

the first visit showing progression according to RECIST 1.1 will be assigned a visit (overall) response of iUPD, regardless of which factors caused the progression.

At this visit, target and non-target lesions identified at baseline by RECIST 1.1 will be assessed as usual.

New lesions will be classified as measurable or non-measurable, using the same size thresholds and rules as for baseline lesion assessment in RECIST 1.1. From measurable new lesions, up to 5 lesions total (up to 2 per organ), may be selected as New Lesions – Target. The sum of diameters of these lesions will be calculated, and kept distinct from the sum of diameters for target lesions at baseline. All other new lesions will be followed qualitatively as New Lesions – Non-target.

Assessment at the Confirmatory Imaging

On the confirmatory imaging, the participant will be classified as progression confirmed (with an overall response of iCPD), or as showing persistent unconfirmed progression (with an overall response of iUPD), or as showing disease stability or response (iSD/iPR/iCR).

Confirmation of Progression

Progression is considered confirmed, and the overall response will be iCPD, if ANY of the following occurs:

- Any of the factors that were the basis for the initial iUPD show worsening
  - For target lesions, worsening is a further increase in the sum of diameters of ≥ 5 mm, compared to any prior iUPD time point
  - For non-target lesions, worsening is any significant growth in lesions overall, compared to a prior iUPD time point; this does not have to meet the "unequivocal" standard of RECIST 1.1
  - For new lesions, worsening is any of these:
    - An increase in the new lesion sum of diameters by ≥ 5 mm from a prior iUPD time point
    - Visible growth of new non-target lesions
    - The appearance of additional new lesions
- Any new factor appears that would have triggered PD by RECIST 1.1

Persistent iUPD

Progression is considered not confirmed, and the overall response remains iUPD, if:

- None of the progression-confirming factors identified above occurs AND

- The target lesion sum of diameters (initial target lesions) remains above the initial PD threshold (by RECIST 1.1)

Additional imaging for confirmation should be scheduled 4 to 8 weeks from the scan on which iUPD is seen. This may correspond to the next visit in the original visit schedule. The assessment of the subsequent confirmation scan proceeds in an identical manner, with possible outcomes of iCPD, iUPD, and iSD/iPR/iCR.

Resolution of iUPD

Progression is considered not confirmed, and the overall response becomes iSD/iPR/iCR, if:

- None of the progression-confirming factors identified above occurs, AND
- The target lesion sum of diameters (initial target lesions) is not above the initial PD threshold.

The response is classified as iSD or iPR (depending on the sum of diameters of the target lesions), or iCR if all lesions resolve.

In this case, the initial iUPD is considered to be pseudo-progression, and the level of suspicion for progression is "reset". This means that the next visit that shows radiographic progression, whenever it occurs, is again classified as iUPD by iRECIST, and the confirmation process is repeated before a response of iCPD can be assigned.

Management Following the Confirmatory Imaging

If repeat imaging does not confirm PD per iRECIST, as assessed by the Investigator, and the participant continues to be clinically stable, study treatment may continue and follow the regular imaging schedule. If PD is confirmed, participants will be discontinued from study treatment.

NOTE: If a participant has confirmed radiographic progression (iCPD) as defined above, but the participant is achieving a clinically meaningful benefit, an exception to continue study treatment may be considered. In this case, if study treatment is continued, tumor imaging should continue to be performed following the intervals as outlined in Section 6.

Detection of Progression at Visits After Pseudo-progression Resolves

After resolution of pseudo-progression (ie, achievement of iSD/iPR/iCR), iUPD is indicated by any of the following events:

- Target lesions
  - Sum of diameters reaches the PD threshold (≥ 20% and ≥ 5 mm increase from nadir) either for the first time, or after resolution of previous pseudo-progression. The nadir is always the smallest sum of diameters seen during the entire trial, either before or after an instance of pseudo-progression.

- Non-target lesions
  - If non-target lesions have never shown unequivocal progression, their doing so for the first time results in iUPD.
  - If non-target lesions had shown previous unequivocal progression, and this progression has not resolved, iUPD results from any significant further growth of non-target lesions, taken as a whole.
- New lesions
  - New lesions appear for the first time
  - Additional new lesions appear
  - Previously identified new target lesions show an increase of ≥ 5 mm in the new lesion sum of diameters, from the nadir value of that sum
  - Previously identified non-target lesions show any significant growth

If any of the events above occur, the overall response for that visit is iUPD, and the iUPD evaluation process (see Assessment at the Confirmatory Imaging above) is repeated. Progression must be confirmed before iCPD can occur.

The decision process is identical to the iUPD confirmation process for the initial PD, except in one respect. If new lesions occurred at a prior instance of iUPD, and at the confirmatory scan the burden of new lesions has increased from its smallest value (for new target lesions, their sum of diameters is ≥5 mm increased from its nadir), then iUPD cannot resolve to iSD or iPR. It will remain iUPD until either a decrease in the new lesion burden allows resolution to iSD or iPR, or until a confirmatory factor causes iCPD.

Additional details about iRECIST are provided in the iRECIST publication [Seymour et al, 2017].

Appendix 5: Adverse Events: Definitions and Procedures for Recording, Evaluating, Follow-up, and Reporting

Definition of AE

AE definition

- An AE is any untoward medical occurrence in a clinical study participant, temporally associated with the use of study intervention, whether or not considered related to the study intervention.
- NOTE: An AE can therefore be any unfavorable and unintended sign (including an abnormal laboratory finding), symptom, or disease (new or exacerbated) temporally associated with the use of a study intervention.
- NOTE: For purposes of AE definition, study intervention (also referred to as Merck product) includes any pharmaceutical product, biological product, vaccine, diagnostic agent, or protocol specified procedure whether investigational or marketed (including placebo, active comparator product, or run-in intervention), manufactured by, licensed by, provided by, or distributed by Merck for human use in this study.

Events meeting the AE definition

- Any abnormal laboratory test results (hematology, clinical chemistry, or urinalysis) or other safety assessments (eg, ECG, radiological scans, vital signs measurements), including those that worsen from baseline, considered clinically significant in the medical and scientific judgment of the investigator.
- Exacerbation of a chronic or intermittent pre-existing condition including either an increase in frequency and/or intensity of the condition.
- New conditions detected or diagnosed after study intervention administration even though it may have been present before the start of the study.
- Signs, symptoms, or the clinical sequelae of a suspected drug-drug interaction.
- Signs, symptoms, or the clinical sequelae of a suspected overdose of either study intervention or a concomitant medication.
- For all reports of overdose (whether accidental or intentional) with an associated AE, the AE term should reflect the clinical symptoms or abnormal test result. An overdose without any associated clinical symptoms or abnormal laboratory results is reported using the terminology “accidental or intentional overdose without adverse effect.”

Events NOT meeting the AE definition

- Medical or surgical procedure (eg, endoscopy, appendectomy): the condition that leads to the procedure is the AE.
- Situations in which an untoward medical occurrence did not occur (social and/or convenience admission to a hospital).
- Anticipated day-to-day fluctuations of pre-existing disease(s) or condition(s) present or detected at the start of the study that do not worsen.
- Surgery planned prior to informed consent to treat a pre-existing condition that has not worsened.

Definition of SAE

If an event is not an AE per definition above, then it cannot be an SAE even if serious conditions are met.

An SAE is defined as any untoward medical occurrence that, at any dose:

a. Results in death

b. Is life-threatening

- The term "life-threatening" in the definition of "serious" refers to an event in which the participant was at risk of death at the time of the event. It does not refer to an event, which hypothetically might have caused death, if it were more severe.

c. Requires inpatient hospitalization or prolongation of existing hospitalization

- Hospitalization is defined as an inpatient admission, regardless of length of stay, even if the hospitalization is a precautionary measure for continued observation. (Note: Hospitalization for an elective procedure to treat a pre-existing condition that has not worsened is not an SAE. A pre-existing condition is a clinical condition that is diagnosed prior to the use of a Merck product and is documented in the participant's medical history.

d. Results in persistent or significant disability/incapacity

- The term disability means a substantial disruption of a person's ability to conduct normal life functions.

- This definition is not intended to include experiences of relatively minor medical significance such as uncomplicated headache, nausea, vomiting, diarrhea, influenza, and accidental trauma (eg, sprained ankle) that may interfere with or prevent everyday life functions but do not constitute a substantial disruption.

e. Is a congenital anomaly/birth defect

- In offspring of participant taking the product regardless of time to diagnosis.

f. Other important medical events

- Medical or scientific judgment should be exercised in deciding whether SAE reporting is appropriate in other situations such as important medical events that may not be immediately life-threatening or result in death or hospitalization but may jeopardize the participant or may require medical or surgical intervention to prevent 1 of the other outcomes listed in the above definition. These events should usually be considered serious.
- Examples of such events include invasive or malignant cancers, intensive treatment in an emergency room or at home for allergic bronchospasm, blood dyscrasias or convulsions that do not result in hospitalization, or development of drug dependency or drug abuse.

Additional Events Reported in the Same Manner as SAE

Additional events that require reporting in the same manner as SAE

In addition to the above criteria, AEs meeting either of the below criteria, although not serious per ICH definition, are reportable to Merck in the same time frame as SAEs to meet certain local requirements. Therefore, these events are considered serious by Merck for collection purposes.

- Is a new cancer (that is not a condition of the study)
- Is associated with an overdose of pembrolizumab

Recording AE and SAE

AE and SAE recording

- When an AE/SAE occurs, it is the responsibility of the investigator to review all documentation (eg, hospital progress notes, laboratory, and diagnostics reports) related to the event.
- The investigator will record all relevant AE/SAE information on the AE CRFs/worksheets at each examination.

- There may be instances when copies of medical records for certain cases are requested by the Merck. In this case, all participant identifiers, with the exception of the participant number, will be blinded on the copies of the medical records before submission to the Merck.
- The investigator will attempt to establish a diagnosis of the event based on signs, symptoms, and/or other clinical information. In such cases, the diagnosis (not the individual signs/symptoms) will be documented as the AE/SAE.

Assessment of intensity/toxicity

- An event is defined as "serious" when it meets at least 1 of the predefined outcomes as described in the definition of an SAE, not when it is rated as severe.

1. The investigator will make an assessment of intensity for each AE and SAE (and other reportable safety event) according to the NCI Common Terminology for Adverse Events (CTCAE), version 5. Any AE that changes CTCAE grade over the course of a given episode will have each change of grade recorded on the AE CRFs/worksheets.
    - Grade 1: Mild; asymptomatic or mild symptoms; clinical or diagnostic observations only; intervention not indicated.
    - Grade 2: Moderate; minimal, local or noninvasive intervention indicated; limiting age-appropriate instrumental activities of daily living (ADL).
    - Grade 3: Severe or medically significant but not immediately life-threatening; hospitalization or prolongation of hospitalization indicated; disabling; limiting self-care ADL.
    - Grade 4: Life threatening consequences; urgent intervention indicated.
    - Grade 5: Death related to AE.

Assessment of causality

1. Did Merck product cause the AE?
2. The determination of the likelihood that Merck product caused the AE will be provided by an investigator who is a qualified physician. The investigator's signed/dated initials on the source document or worksheet that supports the causality noted on the AE form, ensures that a medically qualified assessment of causality was done. This initialed document must be retained for the required regulatory time frame. The criteria below are intended as reference guidelines to assist the investigator in assessing the likelihood of a relationship between the test product and the AE based upon the available information.

3. The following components are to be used to assess the relationship between Merck's product and the AE; the greater the correlation with the components and their respective elements (in number and/or intensity), the more likely Merck product caused the AE:

- **Exposure:** Is there evidence that the participant was actually exposed to Merck product such as: reliable history, acceptable compliance assessment (pill count, diary, etc.), expected pharmacologic effect, or measurement of drug/metabolite in bodily specimen?
- **Time Course:** Did the AE follow in a reasonable temporal sequence from administration of Merck product? Is the time of onset of the AE compatible with a drug-induced effect (applies to studies with investigational medicinal product)?
- **Likely Cause:** Is the AE not reasonably explained by another etiology such as underlying disease, other drug(s)/vaccine(s), or other host or environmental factors.
- **Dechallenge:** Was Merck product discontinued or dose/exposure/frequency reduced?
- If yes, did the AE resolve or improve?
- If yes, this is a positive dechallenge.
- If no, this is a negative dechallenge.
- (Note: This criterion is not applicable if: (1) the AE resulted in death or permanent disability; (2) the AE resolved/improved despite continuation of the Merck product; (3) the study is a single-dose drug study; or (4) Merck product(s) is/are only used 1 time.)
- **Rechallenge:** Was the participant re-exposed to Merck product in this study?
- If yes, did the AE recur or worsen?
- If yes, this is a positive rechallenge.
- If no, this is a negative rechallenge.

(Note: This criterion is not applicable if: (1) the initial AE resulted in death or permanent disability, or (2) the study is a single-dose drug study; or (3) Merck product(s) is/are used only 1 time.)

NOTE: IF A RECHALLENGE IS PLANNED FOR AN AE THAT WAS SERIOUS AND MAY HAVE BEEN CAUSED BY MERCK PRODUCT, OR IF RE-EXPOSURE TO

MERCK'S PRODUCT POSES ADDITIONAL POTENTIAL SIGNIFICANT RISK TO THE PARTICIPANT THEN THE RECHALLENGE MUST BE APPROVED IN ADVANCE BY THE SPONSOR AS PER DOSE MODIFICATION GUIDELINES IN THE PROTOCOL, AND IF REQUIRED, THE INIRB/IEC.

4. **Consistency with study intervention profile:** Is the clinical/pathological presentation of the AE consistent with previous knowledge regarding Merck product or drug class pharmacology or toxicology?

5. The assessment of relationship will be reported on the case report forms/worksheets by an investigator who is a qualified physician according to his/her best clinical judgment, including consideration of the above elements.

6. Use the following scale of criteria as guidance (not all criteria must be present to be indicative of Merck product relationship).

   - Yes, there is a reasonable possibility of Merck product relationship:
   - There is evidence of exposure to the Merck product. The temporal sequence of the AE onset relative to the administration of Merck product is reasonable. The AE is more likely explained by Merck product than by another cause.
   - No, there is not a reasonable possibility of Merck product relationship:
   - Participant did not receive the Merck product OR temporal sequence of the AE onset relative to administration of the Merck product is not reasonable OR the AE is more likely explained by another cause than the Merck product. (Also entered for a participant with overdose without an associated AE.)

7. For each AE/SAE, the investigator must document in the medical notes that he/she has reviewed the AE/SAE and has provided an assessment of causality.

8. There may be situations in which an SAE has occurred and the investigator has minimal information to include in the initial report to the Merck. However, it is very important that the investigator always make an assessment of causality for every event before the initial transmission of the SAE data to Merck.

9. The investigator may change his/her opinion of causality in light of follow-up information and send an SAE follow-up report with the updated causality assessment.

10. The causality assessment is 1 of the criteria used when determining regulatory reporting requirements.

11. For studies in which multiple agents are administered as part of a combination regimen, the investigator may attribute each AE causality to the combination regimen or to a single agent of the combination. In general, causality attribution should be assigned to the combination regimen (ie, to all agents in the regimen). However, causality attribution may be assigned to a single agent if in the investigator's opinion, there is sufficient data to support full attribution of the AE to the single agent.

Follow-up of AE and SAE

- The investigator is obligated to perform or arrange for the conduct of supplemental measurements and/or evaluations as medically indicated to elucidate the nature and/or causality of the AE or SAE as fully as possible. This may include additional laboratory tests or investigations, histopathological examinations, or consultation with other health care professionals.
- New or updated information will be recorded in the CRF.
- The investigator will submit any updated SAE data to Merck within 2 business days but no longer than 3 calendar days of receipt of the information.

Reporting of AEs, SAEs, and Other Reportable Safety Events to the Merck

SAE reports and any other relevant safety information are to be forwarded to the Merck Global Safety facsimile number: +1-215-661-6229

A copy of all 15 Day Reports and Annual Progress Reports is submitted as required by FDA, European Union (EU), Pharmaceutical and Medical Devices agency (PMDA) or other local regulators. Investigators will cross reference this submission according to local regulations to the Merck Investigational Compound Number (IND, CSA, etc.) at the time of submission. Additionally, investigators will submit a copy of these reports to Merck & Co., Inc. (Attn: Worldwide Product Safety; FAX 215-661-6229) at the time of submission to FDA.

nature portfolio

Corresponding author(s): Jung-Yun Lee

Last updated by author(s): Apr 10, 2023

Editorial Policy Checklist

This form is used to ensure compliance with Nature Portfolio editorial policies related to research ethics and reproducibility. For further information, please see our editorial policies site. All relevant questions on the form must be answered.

Competing interests

Policy information about competing interests

In the interest of transparency and to help readers form their own judgements of potential bias, Nature Portfolio journals require authors to declare any competing financial and/or non-financial interest in relation to the work described in the submitted manuscript.

Competing interests declaration

☐ We declare that none of the authors have competing financial or non-financial interests as defined by Nature Portfolio.

☒ We declare that one or more of the authors have a competing interest as defined by Nature Portfolio.

MSD supported the study by providing the study drugs (olaparib and pembrolizumab). JYL, BGK, and JWK received grants from the MSD during the conduct of the study. JYL received grants and personal fees from AstraZeneca, Bei gene, Bergenbio, Clovis Oncology, Immunogen, Janssen, Merck, Novartis, Roche, Seagen, Synthon, and Takeda. BGK received grants from AstraZeneca, Cellid, and Utilex. JWK received personal fees from AstraZeneca, Janssen, Takeda, GSK, Boryung, CMIC, LG Pharma, and Vifor Pharma. DST received consultancy fees from AstraZeneca, Roche, MSD, Merck, Serono, GSK, Eisai, and Genmab; DST is a recipient of research funding and support from MSD, Eisai, AstraZeneca, Bayer, BMS, Karyopharm, and The National Medical Research Council Singapore Clinician Scientist Award – Senior Investigator grant (CSASI21jun-003), and Pangestu Family Foundation Gynaecological Cancer Research Fund. DST has stock ownership: Asian Microbiome Library (AMiLi). NYN received personal fees from AstraZeneca and Pfizer. Otherwise, the authors declare that they have no conflicts of interest.

Authorship

Policy information about authorship

Prior to submission all listed authors must agree to all manuscript contents, the author list and its order and the author contribution statements. Any changes to the author list after submission must be approved by all authors.

☒ We have read the Nature Portfolio Authorship Policy and confirm that this manuscript complies.

Large Language Models (LLMs), such as ChatGPT, do not currently satisfy our authorship criteria. Notably an attribution of authorship carries with it accountability for the work, which cannot be effectively applied to LLMs. Use of an LLM should be properly documented in the Methods section (and if a Methods section is not available, in a suitable alternative part) of the manuscript.

☒ We confirm that the author list of this manuscript does not include any Large Language Models (LLMs).

Policy information about Authorship: inclusion & ethics in global research

All authors are encouraged to provide an "Inclusion & Ethics" statement where relevant.

☒ We have provided an "Inclusion & Ethics" statement.

Data availability

Policy information about availability of data

Data availability statement

All manuscripts must include a data availability statement. This statement should provide the following information, where applicable:

- Accession codes, unique identifiers, or web links for publicly available datasets
- A description of any restrictions on data availability
- For clinical datasets or third party data, please ensure that the statement adheres to our policy

☒ We have provided a full data availability statement in the manuscript.

Mandated accession codes (where applicable)

Confirm that all relevant data are deposited into a public repository and that accession codes are provided.

☐ All relevant accession codes are provided ☐ Accession codes will be available before publication ☐ No data with mandated deposition

Code availability

Policy information about availability of computer code

Code availability statement

For all studies using custom code or mathematical algorithm that is deemed central to the conclusions, the manuscript must include a statement under the heading "Code availability" describing how readers can access the code, including any access restrictions. Code availability statements should be provided as a separate section after the data availability statement but before the References.

☐ We have provided a full code availability statement in the manuscript

Data presentation

For all data presented in a plot, chart or other visual representation confirm that:

n/a	Confirmed	
☐	☒	Individual data points are shown when possible, and always for n ≤ 10
☒	☐	The format shows data distribution clearly (e.g. dot plots, box-and-whisker plots)
☒	☐	Box-plot elements are defined (e.g. center line, median; box limits, upper and lower quartiles; whiskers, 1.5x interquartile range; points, outliers)
☐	☒	Clearly defined error bars are present and what they represent (SD, SE, CI) is noted

Image integrity

Policy information about image integrity

☒ We have read Nature Portfolio's image integrity policy and all images comply.

Unprocessed data must be provided upon request. Please double-check figure assembly to ensure that all panels are accurate (e.g. all labels are correct, no inadvertent duplications have occurred during preparation, etc.).

Where blots and gels are presented, please take particular care to ensure that lanes have not been spliced together, that loading controls are run on the same blot, and that unprocessed scans match the corresponding figures.

Additional policy considerations

Some types of research require additional policy disclosures. Please indicate whether each of these apply to your study. If you are not certain, please read the appropriate section before selecting a response.

Does not apply	Involved in the study	
☒	☐	Macromolecular structural data
☒	☐	Unique biological materials
☒	☐	Research animals and/or animal-derived materials that require ethical approval
☒	☐	Human embryos, gametes and/or stem cells
☐	☒	Human research participants
☐	☒	Clinical data
☒	☐	Archaeological, geological, and palaeontological materials

Human research participants

Policy information about studies involving human research participants

Ethical compliance

☒ We have complied with all relevant ethical regulations and include a statement affirming this in the manuscript.

Ethics committee

Confirm that the manuscript states the name(s) of the board and/or institution that:

☒ Approved the study protocol -OR- ☐ Provided guidelines for study procedures (if protocol approval is not required)

Informed consent

☒ We have obtained informed consent from all participants and this is noted in the manuscript.

Identifiable images

For publication of identifiable images of research participants, confirm that consent to publish was obtained and is noted in the Methods.

Authors must ensure that consent meets the conditions set out in the Nature Portfolio participant release form.

☐ Yes ☒ No identifiable images of human research participants

Clinical studies

Policy information about clinical studies

Clinical trial registration

☒ We have provided the trial registration number from ClinicalTrials.gov or an equivalent agency in the manuscript.

Phase 2 and 3 randomized controlled trials

We have provided the CONSORT checklist with your submission.

☐ Yes ☐ No ☒ Not a phase 2/3 randomized controlled trial

Tumor marker prognostic studies

We have followed the REMARK reporting guidelines.

☒ Yes ☐ No ☐ Not a tumor marker prognostic study

I certify that all the above information is complete and correct.

Typed signature Jung-Yun Lee Date Apr 10, 2023

This checklist template is licensed under a Creative Commons Attribution 4.0 International License, which permits use, sharing, adaptation, distribution and reproduction in any medium or format, as long as you give appropriate credit to the original author(s) and the source, provide a link to the Creative Commons license, and indicate if changes were made. The images or other third party material in this article are included in the article's Creative Commons license, unless indicated otherwise in a credit line to the material. If material is not included in the article's Creative Commons license and your intended use is not permitted by statutory regulation or exceeds the permitted use, you will need to obtain permission directly from the copyright holder. To view a copy of this license, visit http://creativecommons.org/licenses/by/4.0/
