# Supplementary material for: Triplet maintenance therapy of olaparib, pembrolizumab and bevacizumab in women with BRCA wild-type, platinum-sensitive recurrent ovarian cancer: the multicenter, single-arm phase II study OPEB-01/APGOT-OV4
Source: Nat Commun. 2023 Sep 6;14:5476. doi: 10.1038/s41467-023-40829-2 (PMC10482952; doi:10.1038/s41467-023-40829-2)
Supplement: Supplementary file 2 — Reporting Summary [file 41467_2023_40829_MOESM2_ESM.pdf]

nature portfolio

Corresponding author(s): Jung-Yun Lee

Last updated by author(s): Jul 24, 2023

Reporting Summary

Nature Portfolio wishes to improve the reproducibility of the work that we publish. This form provides structure for consistency and transparency in reporting. For further information on Nature Portfolio policies, see our Editorial Policies and the Editorial Policy Checklist.

Statistics

For all statistical analyses, confirm that the following items are present in the figure legend, table legend, main text, or Methods section.

n/a	Confirmed	
☐	☒	The exact sample size (n) for each experimental group/condition, given as a discrete number and unit of measurement
☐	☒	A statement on whether measurements were taken from distinct samples or whether the same sample was measured repeatedly
☐	☒	The statistical test(s) used AND whether they are one- or two-sided<br>*Only common tests should be described solely by name; describe more complex techniques in the Methods section.*
☒	☐	A description of all covariates tested
☒	☐	A description of any assumptions or corrections, such as tests of normality and adjustment for multiple comparisons
☐	☒	A full description of the statistical parameters including central tendency (e.g. means) or other basic estimates (e.g. regression coefficient) AND variation (e.g. standard deviation) or associated estimates of uncertainty (e.g. confidence intervals)
☐	☒	For null hypothesis testing, the test statistic (e.g. F, t, r) with confidence intervals, effect sizes, degrees of freedom and P value noted<br>*Give P values as exact values whenever suitable.*
☒	☐	For Bayesian analysis, information on the choice of priors and Markov chain Monte Carlo settings
☒	☐	For hierarchical and complex designs, identification of the appropriate level for tests and full reporting of outcomes
☒	☐	Estimates of effect sizes (e.g. Cohen's d, Pearson's r), indicating how they were calculated

Our web collection on statistics for biologists contains articles on many of the points above.

Software and code

Policy information about availability of computer code

Data collection	No software was used
Data analysis	Statistical analysis: SAS (version 9.4; SAS institute, Cary, NC, USA); Sequenza-utils (v.3.0.0); scarHRD (R package v.0.1.1).

For manuscripts utilizing custom algorithms or software that are central to the research but not yet described in published literature, software must be made available to editors and reviewers. We strongly encourage code deposition in a community repository (e.g. GitHub). See the Nature Portfolio guidelines for submitting code & software for further information.

Data

Policy information about availability of data

All manuscripts must include a data availability statement. This statement should provide the following information, where applicable:

- Accession codes, unique identifiers, or web links for publicly available datasets
- A description of any restrictions on data availability
- For clinical datasets or third party data, please ensure that the statement adheres to our policy

The full study protocol and statistical analysis plan are available in the Supplementary Note. Data underlying all Figures are provided in the Source Data file. Further data are not publicly available due to patient privacy, but can be accessed on request from the corresponding author Jung-Yun Lee (jungyunlee@yuhs.ac) for 10 years; individual de-identified participant data will be shared for academic research purposes.

Research involving human participants, their data, or biological material

Policy information about studies with human participants or human data. See also policy information about sex, gender (identity/presentation), and sexual orientation and race, ethnicity and racism.

Reporting on sex and gender	Reporting on sex and gender was not relevant in this study.
Reporting on race, ethnicity, or other socially relevant groupings	Reporting on race, ethnicity, or other socially relevant groupings was not relevant in this study.
Population characteristics	BRCA wildtype patients with platinum-sensitive recurrent ovarian cancer receiving triplet maintenance with olaparib, pembrolizumab, and bevacizumab. The median age was 61 (range: 43-78).
Recruitment	Eligible patients were ≥ 20 years of age, with an Eastern Cooperative Oncology Group performance status of 0 or 1, histologically confirmed epithelial ovarian cancer, and lacking germline and/or tumor BRCA mutations. With respect to histology, patients with high-grade predominantly serous, endometrioid, carcinosarcoma, mixed Mullerian with high-grade serous components, clear cell, or low-grade serous ovarian cancer, primary peritoneal cancer, or fallopian tubal cancer were considered. A cap of 8 patients was applied for clear cell carcinoma; mucinous carcinoma could be enrolled. Patients had received two previous courses of platinum-containing therapy and showed platinum-sensitive disease (platinum-free interval of ≥ six months) following their penultimate platinum course, along with a complete response (CR) or PR to their most recent platinum course; they were enrolled in the study within eight weeks of completing their final platinum regimen, regardless of prior PARP inhibitor or bevacizumab use but had to be immunotherapy naïve. Based on this inclusion criteria, we have offered all eligible patients to participate in this study. We did not advertise or target a specific group of patients to participate.
Ethics oversight	The trial was conducted in accordance with the Declaration of Helsinki and the Guidelines for Good Clinical Practice. The trial was approved by the institutional review board of each institution (Severance Hospital: 4-2020-0386; Seoul National University Hospital: H-2101-017-1186; Samsung Medical Center: SMC 2020-08-078; National Cancer Center: NCC2021-0069; National University Cancer Institute: 2020/01198). Written informed consent was obtained from all participants before study enrollment.

Note that full information on the approval of the study protocol must also be provided in the manuscript.

Field-specific reporting

Please select the one below that is the best fit for your research. If you are not sure, read the appropriate sections before making your selection.

☒ Life sciences ☐ Behavioural & social sciences ☐ Ecological, evolutionary & environmental sciences

For a reference copy of the document with all sections, see nature.com/documents/nr-reporting-summary-flat.pdf

Life sciences study design

All studies must disclose on these points even when the disclosure is negative.

Sample size	The study was conducted using Simon's two-stage optimal design with assumptions concerning the estimated progression-free survival (PFS) rate in ovarian cancer. As the benchmark for the null hypothesis, we chose the GOG 213 study, which investigated chemotherapy plus bevacizumab followed by bevacizumab maintenance regardless of BRCA mutations. Recognizing the conceivable differences between GOG 213 and our trial, which focuses on the maintenance therapy, we used the best approximation from GOG 213 by considering the chemotherapy time window, because of the lack of data on studies with bevacizumab maintenance in patients responding to chemotherapy. Thus, based on the current standard of care and the best approximation from GOG 213, the rate of patients with a disease-free state at 6 months was expected to be 50% with bevacizumab maintenance. Moreover, the HR of adding maintenance therapy with a triplet combination (PARP inhibitor, immune checkpoint inhibitor (ICI), and antiangiogenic therapy) was assumed to be 0.5, equivalent to a PFS rate of 70.7 %. The null hypothesis for this study would be a 6-month PFS rate of 50%, and the alternative hypothesis of interest would be a 6-month PFS rate of 70%. Using Simon's two-stage optimal design at a one- sided 5% level of significance and 80% power, 39 patients were included in this study. In the first stage, 22 patients would be enrolled; if 10 or more progressive diseases (PDs) were observed, the trial would be terminated. Else, the trial would continue to the second stage. The null hypothesis would be rejected if the total number of PDs was less than 15. Considering loss to follow-up, the 44 patients would be studied.
Data exclusions	Patients with specific histology subtypes (mucinous, germ cell, or borderline) or deleterious germline or somatic BRCA mutation were excluded. Patients were also excluded based on previous medical conditions such as non-infectious pneumonitis, myelodysplastic syndrome (MDS) or acute myeloid leukemia (AML), autoimmune disease, or additional malignancy requiring treatment. The detailed exclusion criteria are mentioned in the study protocol.
Replication	This is a clinical trial (investigator-initiated, open-label, single-arm, Phase II study), and this study is equivalent to one replicate. Findings from this study can form the basis for future Phase III trials.
Randomization	Based on the study conception, this study was single-arm.
Blinding	Based on the study conception, this study was open-label.

Reporting for specific materials, systems and methods

We require information from authors about some types of materials, experimental systems and methods used in many studies. Here, indicate whether each material, system or method listed is relevant to your study. If you are not sure if a list item applies to your research, read the appropriate section before selecting a response.

Materials & experimental systems

n/a	Involved in the study	
☐	☒	Antibodies
☒	☐	Eukaryotic cell lines
☒	☐	Palaeontology and archaeology
☒	☐	Animals and other organisms
☐	☒	Clinical data
☒	☐	Dual use research of concern
☒	☐	Plants

Methods

n/a	Involved in the study	
☒	☐	ChIP-seq
☒	☐	Flow cytometry
☒	☐	MRI-based neuroimaging

Antibodies

Antibodies used	Immunohistochemistry (IHC) was performed using a Ventana Benchmark XT automated stainer (Ventana Medical Systems, Arizona, United States) with antibodies against PD-L1 (pre-diluted, clone 22C3, DAKO, Glostrup, Denmark). PD-L1 expression in the tumor cell membrane and the membrane and/or cytoplasm of tumor-associated mononuclear inflammatory cells was scored. The combined positive score (CPS) was defined as the total number of tumors and immune cells stained with PD-L1 divided by the number of all viable tumor cells and then multiplied by 100.
Validation	This antibody is commercially available and was used for applications validated by the manufacturer.

Clinical data

Policy information about clinical studies

All manuscripts should comply with the ICMJEguidelines for publication of clinical research and a completedCONSORT checklist must be included with all submissions.

Clinical trial registration	NCT04361370
Study protocol	Full study protocol is supplied in the supplementary.
Data collection	This investigated-initiated, multi-center, single-arm, open-label, Phase II study was conducted in five medical centers across Korea and Singapore. Between October 20, 2020, and March 22, 2022, 44 patients with platinum-sensitive recurrent ovarian cancer who came to Severance Hospital of Yonsei University, Seoul National University Hospital, Samsung Medical Center of Sungkyunkwan University, National Cancer Center in Korea, and National University Cancer Institute of Singapore were screened and enrolled. Clinical data were entered into a web-based electronic case report form (CRF) by the clinical site staff. The origin of the data was the medical records, charts, and reports available at each clinical sites where the patients were treated.
Outcomes	The primary endpoint was the 6-month PFS rate. PFS was defined as the time from the start of treatment to the first documented sign of disease progression or death from any cause. The secondary endpoints included PFS, and safety. Overall survival (OS) was defined as the time from the first treatment to death from any cause.
